# Air toxics and the risk of autism spectrum disorder: the results of a population based case–control study in southwestern Pennsylvania

**DOI:** 10.1186/s12940-015-0064-1

**Published:** 2015-10-06

**Authors:** Evelyn O. Talbott, Lynne P. Marshall, Judith R. Rager, Vincent C. Arena, Ravi K. Sharma, Shaina L. Stacy

**Affiliations:** Department of Epidemiology, University of Pittsburgh, Graduate School of Public Health, 130 DeSoto Street, A526 Crabtree, Pittsburgh, PA 15261 USA; Department of Biostatistics, University of Pittsburgh, Graduate School of Public Health, Pittsburgh, PA USA; Department of Behavioral and Community Health Scienc, University of Pittsburgh, Graduate School of Public Health, Pittsburgh, PA USA; Department of Environmental and Occupational Health, University of Pittsburgh, Graduate School of Public Health, Pittsburgh, PA USA

**Keywords:** Air toxics, Autism spectrum disorder, Case–control study, Chromium, Geographic information system, Styrene

## Abstract

**Background:**

Autism spectrum disorders (ASD) constitute a major public health problem affecting one in 68 children. There is little understanding of the causes of ASD despite its serious social impact. Air pollution contains many toxicants known to have adverse effects on the fetus. We conducted a population based case–control study in southwestern Pennsylvania to estimate the association between ASD and 2005 US EPA modeled NATA (National Air Toxics Assessment) levels for 30 neurotoxicants.

**Methods:**

A total of 217 ASD cases born between 2005 and 2009 were recruited from local ASD diagnostic and treatment centers. There were two different control groups: 1) interviewed controls (*N* = 224) frequency matched by child’s year of birth, sex and race with complete residential histories from prior to pregnancy through the child’s second birthday, and 2) 5,007 controls generated from a random sample of birth certificates (BC controls) using residence at birth. We used logistic regression analysis comparing higher to first quartile of exposure to estimate odds ratios (ORs) and 95 % confidence intervals (CI), adjusting for mother’s age, education, race, smoking status, child’s year of birth and sex.

**Results:**

Comparing fourth to first quartile exposures for all births, the adjusted OR for styrene was 2.04 (95 % CI = 1.17–3.58, *p* = 0.013) for the interviewed case–control analysis and 1.61 (95 % CI = 1.08-2.40, *p* = 0.018) for the BC analysis. In the BC comparison, chromium also exhibited an elevated OR of 1.60 (95 % CI = 1.08-2.38, *p* = 0.020), which was similarly elevated in the interviewed analysis (OR = 1.52, 95 % CI = 0.87–2.66). There were borderline significant ORs for the BC comparison for methylene chloride (OR = 1.41, 95 % CI = 0.96–2.07, *p* = 0.082) and PAHs (OR = 1.44, 95 % CI = 0.98–2.11, *p* = 0.064).

**Conclusions:**

Living in areas with higher levels of styrene and chromium during pregnancy was associated with increased risk of ASD, with borderline effects for PAHs and methylene chloride. These results are consistent with other studies. It is unclear, however, whether these chemicals are risk factors themselves or if they reflect the effect of a mixture of pollutants. Future work should include improved spatiotemporal estimates of exposure to air toxics, taking into account the dynamic movement of individuals during daily life.

**Electronic supplementary material:**

The online version of this article (doi:10.1186/s12940-015-0064-1) contains supplementary material, which is available to authorized users.

## Background

Autism spectrum disorders (ASD) constitute a major public health problem, affecting approximately one in every 68 children and their families [1]. ASDs are brain development disorders usually diagnosed in childhood and characterized by impaired social interaction and communication, and by restricted and repetitive behaviors [2]. In many cases, individuals with ASD cannot live independently and may require lifetime assistance from a family member or caregiver [3]. The families of children with ASD are at an increased risk for mental and physical health problems, parenting stress, financial strain, divorce, and overall lower family well-being [3]. Because ASDs are lifelong conditions for which there is no cure and for which treatment options are limited, there is a need to explore additional potential risk factors for these disorders.

Despite its serious social impact [4], the causes of autism are poorly understood, but both genetic (10–20 %) and environmental factors are thought to be involved. Researchers have noted several risk factors related to ASD including advanced maternal and/or paternal age, maternal smoking history, low birth weight, short gestational duration, and hypoxia during childbirth [5]. In addition, environmental factors, including constituents of air pollution, have been found to be related to an increased risk of autism [6, 7]. These include: PM_2.5_, ozone, NO_2_, pesticides, heavy metals, solvents, and diesel exhaust [7–16].

Although there are differences in the time periods of the investigations, geographical regions under study and the exposure assessments, a growing number of studies suggest a potential role of ASD and air pollution. Recently, several investigations have focused on ASD risk and ambient criteria air pollutants (traffic related and/or from industry) [12–16]. Volk et al. (2011, 2013) examined the relationship between traffic-related air pollution and autism in California in two population based case–control studies from the Childhood Autism Risks from Genetics and the Environment (CHARGE) study. The first reported increased risk among those living within 1,000 feet of a major freeway [12], and the second found that increased autism risk was associated with exposure to model-based indicators of traffic-related air pollutant (TRP) mixture as well as regional measures of NO_2_, PM_2.5_, and PM_10_ [13]. Becerra et al. also noted increased ASD risk per interquartile range increases of ozone, PM_2.5_, NO, and NO_2_ [14].

To date, there have been three case–control studies of ASD risk that utilized data from the National Air Toxics Assessment (NATA) to examine exposure to specific neurological toxicants, developmental toxicants, or suspected endocrine disruptors during pregnancy [17–19]. NATA is a US EPA developed database with modeled annual average concentrations of hazardous air pollutants (HAPs) at the state, county, and census tract levels. To model annual average ambient concentrations of air toxics for each census tract, NATA assumes Gaussian air dispersion and uses overlapping spatial grids. Model inputs include locations and rates of pollutant emissions from point, non-point, and mobile sources, as well as secondary-pollutant formation and meteorological data. NATA assessments have been released every three years from 1996 until 2005 [20]. These studies were conducted in various regions of the US and involved time periods between 1990 and 2002. In their investigations, Windham et al. and Kalkbrenner et al. reported that increased ASD risk was associated with higher levels of exposure to groups of heavy metals and chlorinated solvents [19] and to the individual compounds methylene chloride, quinolone, and styrene [17]. Roberts et al. (2013) also noted associations between ASD risk and exposure to overall metals and methylene chloride, as well as lead, manganese and diesel particulate matter [18].

Von Ehrenstein et al. (2014) considered the risk of autistic disorder related to ground level measured exposure to monitored ambient air toxics from urban emissions during the pregnancy period [21]. Birth records were linked to California Department of Developmental Services records of children diagnosed with primary ASD compared to all other births as controls (*N* = 768). ASD risk was increased per interquartile range increase in average concentrations during pregnancy for several correlated toxics, including 1,3-butadiene, meta/para xylene, other aromatic solvents, PERC and formaldehyde, after adjustment for mother’s age, race/ethnicity, education, insurance type, parity, sex and birth year [21].

Given these few epidemiological investigations and the heavy industrial background of southwestern Pennsylvania, the objective of the current study was to conduct an exploratory case–control study in this area to assess if neurotoxic air pollutants, as modeled by NATA, were associated with the risk of ASD. We focused on suspect agents found in the previous three investigations which used NATA data and evaluated whether there was an increased risk of ASD associated with higher exposures to these *a priori* agents: arsenic, cadmium, chromium, lead, mercury, manganese, nickel, styrene, trichloroethylene, methylene chloride, vinyl chloride, and diesel particulate matter. Further, we examined additional compounds identified as neurotoxicants, endocrine disruptors, or developmental toxicants by the US EPA [20].

## Methods

This study was approved by the University of Pittsburgh Institutional Review Board (IRB number PRO10010240).

### Ascertainment of cases

Cases of ASD for this study were children born between January 1, 2005 and December 31, 2009 in Allegheny, Armstrong, Beaver, Butler, Washington, or Westmoreland County in southwestern Pennsylvania and who were currently residing in the six-county area. Our goal was to enroll approximately half of the prevalent cases among this birth cohort. Based on 23,399 births in 2007 in the six county area [22] and a prevalence of ASD of 6 per 1,000 (one in 166), we estimated that 140–141 children per year would be diagnosed with ASD in the study area. We anticipated enrolling half (70–71) of these children each year to result in 250 cases during the 3½ year period of the study.

There is no autism registry in Pennsylvania, and therefore no centralized agency that could be accessed for permission to contact parents of children with ASD for the purposes of conducting a study. Our investigation used an extensive outreach campaign to recruit ASD cases from a combination of 1) ASD specialty diagnostic and treatment centers, 2) private pediatric and psychiatry practices, 3) school-based special needs programs (starting at age 5), and 4) autism support groups. Per our IRB guidelines, we were not allowed to directly contact parents of children with ASD. Therefore, we provided informational packets to these agencies and organizations with a letter, contact sheet and pre-addressed envelope to be returned to our office, and these agencies mailed the information to families with ASD. When a contact sheet was returned, we were permitted to contact the mother to describe the study and request consent to participate.

A case of ASD was defined as any child 1) who scored a 15 or above on the Social Communication Questionnaire (SCQ), a positive screen for the presence of autistic features, and 2) for whom there was written documentation, including ADOS or other test results, of a diagnosis of an ASD from a child psychologist or psychiatrist. Cases were not included in the study if the child was adopted, parents were not English speaking, or a parent was not available for interview. A total of 217 cases were consented and interviewed for the study.

### Ascertainment of controls

The study was designed to have two different sets of controls. The first control group (interviewed controls) was recruited from a random selection of 5007 births weighted by sex (4:1 male:female) from the Pennsylvania Department of Health (PA DOH) state birth registry files for 2005 to 2009 in the six-county area. Interviewed controls were frequency matched to the cases on year of birth, sex, and race. We recruited through a direct letter appeal signed by the Pennsylvania Secretary of Health. The rules for both the PADOH IRB and that of the University of Pittsburgh mandated that there would be no direct contact with potential controls, except for the opportunity to return an envelope and contact sheet indicating refusal to be in the study or an indication that the parent was interested in enrolling his or her child in the study by providing contact information. We requested a postal service return to sender, address correction requested. However, since we used an address from the birth certificate that was several years old, it is likely that many of the letters were never delivered to the intended resident or were simply ignored, making it difficult to determine a true response rate.

After we obtained informed consent, parents were screened for inclusion criteria and administered the SCQ for their child. Children with an SCQ >15 or with a reported diagnosis of ASD were not included as controls. Other exclusion criteria were the same as those for cases. The first control group consisted of 226 eligible controls that were consented and interviewed.

For each of the cases and the interviewed controls, a personal interview with the mother was conducted by trained interviewers using a structured questionnaire, adapted from the CDC’s Study to Explore Early Development (SEED). The questionnaire included parental demographic and socioeconomic information, a detailed residential history, maternal and paternal occupational history, family history of ASD, smoking history, maternal reproductive and pregnancy history, and child’s medical history. Data was obtained on all residential addresses and the corresponding start and end dates that the mother/child lived at those addresses from three months prior to last menstrual period (LMP) until the child’s second birthday.

The second control group (birth certificate or BC controls) consisted of a random sample of births occurring from 2005 to 2009 for the six county area of study, weighted with a male to female ratio of 4:1 and year of birth. Birth certificate information on the cases and controls, consisting of residence at birth, age of mother, smoking history, maternal education, race and other infant characteristics, was then used for the second case–control analysis. Of the total sample of 5,007 birth certificates, 16 were identified as being in our case (ASD) population and were removed from the control group.

### Exposure assessment

Exposure to ambient hazardous air pollution concentrations was estimated using modeled data from the 2005 NATA assessment. The 2005 NATA estimates are an annual average by census tract and were downloaded from the US EPA website (http://www.epa.gov/ttn/atw/nata2005/tables.html accessed April 16, 2014). Out of the 177 air toxics available through NATA, we examined the distribution, variability, and correlations of 37 air toxics characterized as having neurological, developmental or endocrine-disrupting effects by one of the previous studies [17–19] or the US EPA [20]. Seven chemicals (carbon tetrachloride, chloroform, ethylene dibromide, ethylene dichloride, hexachlorobenzene, methyl chloride, and PCBs) were excluded from further analysis due to little diversity in their distributions within the six-county area, leaving a total of 30 NATA compounds for analysis.

For the analyses of the interviewed cases and controls, the residential addresses obtained during the interview were geocoded to an X, Y coordinate using ArcGIS (version 10.1; ESRI Inc., Redlands, CA) and verified manually. When an address could not be successfully geocoded in ArcGIS, other methods were used, including MapQuest Latitude/Longitude Finder (http://developer.mapquest.com/web/tools/lat-long-finder). Year 2000 census tracts (11 digit FIPS codes) for each address were assigned using ArcGIS 10.1, linking to 2009 Tiger Line files for the 2000 United States census. We calculated person-specific exposure estimates for each of the air toxic compounds, taking into account the locations of and changes in residence and the time spent at each residence. For each child, average exposure estimates were computed for the time periods of pregnancy, first year of life, and second year of life. Two participants who lived at a residence outside of the United States for which no NATA data was available were excluded from analysis, leaving an analytic group of 217 cases and 224 controls.

For the birth certificate data analysis, NATA concentrations were linked to census tract of residence at birth. All births that could be linked to a PA DOH birth certificate either contained the census tract of birth or the zip code of birth. When only zip code was provided, the 2010 ZCTA shapefile was used to calculate the geographical center of each zip code in ArcGIS 10.2. Then, each ZCTA centroid was spatially linked to the 2000 census tract that contains it. Of the 217 cases, one of the births could not be linked to its birth certificate, 187 had a census tract on the birth certificate, and 29 only had a zip code of birth. Of the 5,007 potential controls, 16 births were actually in our case population, 4,194 had a census tract on the birth certificate, and 797 only had a zip code of birth. However, 20 control births could not be assigned a NATA exposure: Eighteen could not be linked to a census tract as the documented zip code was not in the 2010 ZCTA shapefile, and two had census tracts documented on the birth certificate that did not match a census tract in the 2005 NATA database. Therefore, the final population in the analysis of BC controls was 216 cases and 4,971 controls.

### Statistical analysis

We used logistic regression to investigate the association between exposure to NATA air pollutants and the risk of autism spectrum disorder. In order to calculate individual odds ratios, quartile cut points were calculated for each of the 30 NATA pollutants. These were based on the distribution among the interviewed controls for use in each respective case–control comparison. The three highest quartiles were individually compared to the lowest quartile. For the interviewed cases and controls, separate logistic regression models were conducted for each pollutant during the pregnancy period and secondarily for the first and second year of life. For the birth certificate control comparison, only residence at the time of birth was available. All analyses were adjusted for maternal age, education, race, smoking, child’s birth year and child’s sex.

In addition to examining compounds individually, we also grouped compounds by structural properties into three classifications: metals excluding selenium (arsenic, cadmium, chromium, lead, manganese, mercury, and nickel), aromatic solvents (benzene, ethyl benzene, styrene, toluene and xylenes), and chlorinated solvents (methylene chloride, perchloroethylene, trichloroethylene, trichloroethane, and vinyl chloride). Index scores were computed for each of the structural groups of metals, aromatic solvents, and chlorinated solvents by summing the quartiles for the compounds in each group. Similar to what was done for the individual compounds, quartile cut points of these scores were calculated based on the distribution of the index scores among the interviewed controls. Logistic regression models comparing highest quartiles to the lowest quartile were conducted for each of the indices for both the interviewed and BC comparisons, controlling for mother’s age, education, race, smoking, child’s year of birth and sex. IBM SPSS Statistics 20 and 22 were used for all analyses. No formal adjustment was made for multiple comparisons.

Additionally, we noted a significantly higher number of multiple births reported among cases compared to controls (8.4 % among the cases; 4.0 % and 3.8 % among the interviewed and birth certificate control groups, respectively). As there is a high rate of prematurity and other problems associated with multiple births, we conducted a sensitivity analysis with and without the inclusion of multiple births for both case–control comparisons.

One of the last steps involved a backward multiple logistic analysis of all agents identified as significant in either case–control comparison with adjustment for mother’s age, race, education, smoking, child’s birth year, and child’s sex. This was done in order to consider the most significant effects of NATA compounds while controlling for the same covariates that were used in the previous logistic regression models for individual pollutants.

Finally, air toxics are often correlated with each other, and people are often simultaneously exposed to a complex mixture of air pollutants. In our study, the Spearman correlation matrix revealed that many of the air toxics were highly correlated (*p* < 0.01). Similar to the methodology detailed by von Ehrenstein et al [21], we conducted a factor analysis to further examine the correlation structure of our set of 30 air toxics. Factors were extracted using Principal Component Analysis (PCA) and rotated using varimax rotation. The eigenvalue >1 rule was used to determine which factors to retain [21].

## Results

A total of 299 families returned a contact sheet and were initially consented and screened for this study (see Additional file 1: Figure S1). Of these, 56 did not meet case inclusion criteria. An additional seven were no longer interested in participating when contacted, and 19 failed to complete the entire interview. The final number of interviewed cases was *n* = 217. These individuals met the requirements for SQL screening and confirmation of diagnosis. The referring sources of the 217 ASD cases were as follows: 116 (49 %) were diagnosed from specialty ASD diagnostic and treatment centers/clinics, 47 (21.6 %) were recruited through private pediatric psychology/psychiatry practices/University, 43 (20 %) were recruited through school-based special needs programs, and 11 (5.1 %) were recruited through autism support groups or our website.

During recruitment, informational letters and contact sheets with return envelopes were mailed to 3,254 households (65 % of the random sample of 5,007 potential controls) (see Additional file 1: Figure S2). There were 369 mailings for which we received an address correction and re-mailed the informational packets. Of the 3,254 prospective participants who were sent letters, 143 were returned as a refusal and 2,861 were either return to sender, forwarding order expired, or no response. A total of 250 potential controls returned contact sheets and consent forms. Of these, 24 were ineligible or unable to be further contacted. The final number of eligible controls who were interviewed was 226.

Characteristics of the study population and estimated exposure to NATA air toxics are presented in Tables 1 and 2. Case–control information is presented according to the information source used for each analysis. In Table 1, information obtained from the interview is presented for the 217 cases and 226 interviewed controls, with the exception of information on LBW (low birth weight, <2500 g) and PTB (preterm birth, <37 weeks gestation), which was obtained from the birth certificate. Information from the birth certificate is presented for the 216 cases for which birth certificate information was available and the 4,971 birth certificate controls.

**Table 1:** Characteristics of ASD Cases and Controls (interviewed information versus information from birth certificate)^a^

	Interviewed		Birth Certificate	
Characteristic	Cases (*n* = 217)	Controls (*n* = 226)	Cases (*n* = 216)	Controls (*n* = 4,971)
	N (%)	N (%)	N (%)	N (%)
Sex:				
Male	169 (77.9)	175 (77.4)	168 (77.8)	3,980 (80.1)
Female	48 (22.1)	51 (22.6)	48 (22.2)	991 (19.9)
Year of birth – n(%)				
2005	43 (19.8)	53 (23.5)	43 (20.0)	1,153 (23.2)
2006	59 (27.2)	44 (19.5)	59 (27.3)	1,151 (23.2)
2007	48 (22.1)	46 (20.4)	48 (22.2)	1,066 (21.4)
2008	37 (17.1)	41 (18.1)	36 (16.7)	888 (17.9)
2009	30 (13.8)	42 (18.6)	30 (13.9)	713 (14.3)
County at birth – n(%)				
Allegheny	131 (60.4)	133 (58.8)	129 (59.7)	2,883 (58.0)
Armstrong	7 (3.2)	2 (0.9)	7 (3.2)	151 (3.0)
Beaver	7 (3.2)	18 (8.0)	7 (3.2)	393 (7.9)
Butler	17 (7.8)	19 (8.4)	18 (8.3)	409 (8.2)
Washington	22 (10.1)	24 (10.6)	21 (9.7)	448 (9.0)
Westmoreland	33 (15.2)	30 (13.3)	34 (15.7)	687 (13.8)
Maternal age (SD) – mean^b^	30.4(5.4)	31.8 (4.7)	30.4 (5.4)	28.5 (6.0)
Paternal age (SD) – mean^2^	32.6 (6.0)	33.6 (5.9)	32.8 (5.8)	31.4 (6.6)
Mother race^d^				
White	194 (89.4)	219 (96.9)	196 (90.7)	4,055 (81.9)
Black	15 (6.9)	4 (1.8)	16 (7.4)	692 (14.0)
Other	2 (0.9)	2 (0.9)	4 (1.9)	205 (4.1)
Mother’s education^e^				
< High school graduate	3 (1.4)	2 (0.9)	6 (2.8)	475 (9.6)
High school graduate and some college	95 (43.8)	46 (20.4)	92 (42.6)	2,536 (51.3)
≥ College graduate	119 (54.9)	178 (78.7)	118 (54.6)	1,937 (39.2)
Low birth weight (<2500 g)^f,i^ (based on birth certificate)	23 (10.7)	9 (4.0)	23 (10.6)	341 (6.9)
Preterm birth (< 37 weeks)^g,j^ (based on birth certificate)	31 (14.8)	20 (9.0)	31 (14.8)	514 (10.6)
Multiple births	18 (8.4)	9 (4.0)	18 (8.4)	189 (3.8)
Maternal Smoking during pregnancy or in 3 months prior to pregnancy^h^				
Yes	54 (24.9)	24 (10.6)	30 (13.9)	1,165 (23.9)
No	163 (75.1)	202 (89.4)	185 (86.0)	3,714 (76.1)

^a^Includes multiples births

^b^Missing 2

^c^Missing 632

^d^Missing 19

^e^Missing 23

^f^Missing 13

^g^Missing 119

^h^Missing 92 controls and 1 case

^i^Missing 1 case

^j^Missing 4 cases and 6 controls

Interview missing information

**Table 2:** Single pollutant percentiles of concentration (ng/m^3^), full pregnancy period, for ASD interviewed cases (*n* = 217) and controls (*n* = 224) and birth certificate cases (*n* = 216) and controls (*n* = 4,971)

Class	Compounds (Concentration In ng/m^3^)	Cases (217)			Controls (224)			Cases (216)			Second Controls (4,971)		
		25	50	75	25	50	75	25	50	75	25	50	75
Metal Compounds	Arsenic	1.03	1.14	1.33	0.98	1.11	1.30	1.02	1.13	1.33	1.00	1.13	1.35
	Cadmium	0.13	0.15	0.18	1.13	0.15	0.18	0.13	0.15	0.18	0.12	0.15	0.19
	Chromium	1.59	1.80	2.40	1.54	1.74	2.06	1.58	1.79	2.30	1.55	1.79	2.18
	Mercury	0.04	0.05	0.08	0.04	0.06	0.08	0.04	0.05	0.08	0.04	0.06	0.08
	Manganese	1.71	2.10	2.38	1.78	2.08	2.27	1.66	2.07	2.36	1.71	2.08	2.30
	Nickel	0.79	0.91	1.06	0.82	0.92	1.02	0.78	0.91	1.04	0.79	0.92	1.07
	Lead	3.40	3.83	4.31	3.29	3.84	4.25	3.36	3.79	4.34	3.27	3.81	4.42
	Selenium	0.35	0.45	0.52	0.38	0.47	0.55	0.36	0.46	0.53	0.32	0.46	0.53
Aromatic Solvents	Benzene	870.78	1102.56	1283.58	823.05	1069.07	1252.91	883.78	1091.58	1289.37	823.50	1082.76	1313.56
	Ethyl benzene	98.16	128.00	188.42	87.25	123.49	181.64	97.35	124.72	188.50	85.25	125.73	196.10
	Styrene	25.22	33.46	51.39	22.82	30.82	40.34	24.43	32.46	50.52	23.67	33.53	48.71
	Toluene	1588.52	2034.18	2501.53	1494.39	2008.97	2439.93	1637.27	2027.70	2536.57	1470.03	2032.16	2553.37
	Xylenes	430.15	591.08	864.09	385.16	536.36	816.75	429.88	583.81	865.79	382.52	588.09	897.42
Chlorinated Solvents	Methylene chloride	241.83	266.47	272.42	241.57	265.99	270.82	244.06	266.47	272.48	239.39	266.53	272.60
	1,1,1-Trichloroethane	215.38	230.86	248.70	210.65	227.25	245.87	215.16	230.84	246.74	211.62	230.70	250.15
	Perchloroethylene	99.77	216.12	266.51	100.31	215.36	262.03	100.08	214.81	267.36	93.71	209.17	265.98
	Trichloroethylene	70.59	74.75	82.92	70.04	73.50	81.64	70.55	74.33	82.46	70.03	74.93	84.26
	Vinyl chloride	0.06	0.09	0.12	0.06	0.09	0.11	0.06	0.09	0.12	0.06	0.09	0.12
Other HAPs	Hydrazine	0.04	0.06	0.06	0.06	0.06	0.06	0.04	0.06	0.06	0.04	0.06	0.07
	PAHs	8.85	12.09	15.87	8.65	11.02	14.60	8.85	12.41	15.72	8.34	12.19	15.70
	Diesel PM	291.11	427.28	660.14	255.12	399.98	589.45	296.99	411.34	614.12	253.58	417.56	666.33
	Allyl chloride	<0.01	<0.01	<0.01	<0.01	<0.01	<0.01	<0.01	<0.01	<0.01	<0.01	<0.01	<0.01
	Carbon disulfide	1.60	2.35	3.35	1.46	2.35	2.97	1.55	2.35	3.38	1.55	2.38	3.27
	Cresol	3.38	4.11	4.56	3.33	4.02	4.49	3.44	4.11	4.52	3.30	4.08	4.58
	Cumene	0.95	1.38	1.68	0.91	1.20	1.65	0.94	1.37	1.66	0.90	1.32	1.69
	Cyanide	48.02	68.14	93.24	44.19	59.91	88.38	45.72	67.14	93.11	44.92	67.32	95.47
	2,4-Dinitrotoluene	<0.01	0.01	0.01	<0.01	0.01	0.01	<0.01	0.01	0.01	<0.01	0.01	0.01
	Ethylene oxide	3.48	3.98	4.64	3.37	3.96	4.54	3.48	3.94	4.57	3.40	3.97	4.77
	Hexane	100.15	137.65	201.95	88.07	128.22	190.27	99.95	131.03	198.83	84.67	133.28	205.30
	Methanol	197.44	273.58	408.06	183.92	253.36	348.18	191.54	272.65	396.41	189.02	277.16	403.32

The majority of both cases and controls were born in Allegheny County, the most populated of the six county areas. Mothers of ASD cases were slightly younger overall than mothers of interviewed controls, but were older than the BC controls. More than half of the mothers of both the interviewed cases and controls reported on interview that they had a college degree or greater; the percent of mothers with a college degree was significantly higher among mothers of interviewed controls compared to cases. The mothers of the 4,971 birth certificate controls, however, had less educational attainment than the mothers of cases (birth certificate information). Cigarette smoking at any time from three months prior to pregnancy until birth was higher among mothers of interviewed ASD cases compared to mothers of interviewed controls. Using the birth certificate information, mothers of cases reported smoking less prior or during pregnancy compared to their birth certificate control counterparts. Preterm births among ASD cases were significantly greater compared to both control groups, largely due to a greater percentage of multiple births (see Table 1).

Table 2 presents the single pollutant 25^th^, 50^th^, and 75^th^ percentiles of concentrations of selected NATA hazardous air pollutants for 1) interviewed ASD cases and controls during the pregnancy period, and for 2) cases and the BC controls using the residence at the time of birth from the birth certificate. As the exposure was assigned differently for the two groups of cases and controls (using the residential history during the entire pregnancy period from the interview versus the residence at birth from the birth certificate), we compared the distributions. In the comparison of the two data sources for the cases, we found no significant differences (Mann Whitney test). A comparison between the two control groups (interviewed versus BC), however, showed significant differences in the distributions for selenium, styrene, and cyanide; all were higher for the second control group.

### Logistic regression results

In the unadjusted logistic regression analysis of the interviewed case–control comparison (not shown), there were statistically significant odds ratios for fourth compared to first quartile of styrene and chromium. PAHs and methanol were of borderline significance for fourth compared to first quartile. For the birth certificate unadjusted analyses (also not shown), associations were statistically significant for toluene third versus first and styrene second versus first quartile. Results were of borderline significance (*p* < 0.10) for second versus first quartile for lead, PAHs, and diesel PM, and third versus first quartile for benzene.

**Fig. 1:**
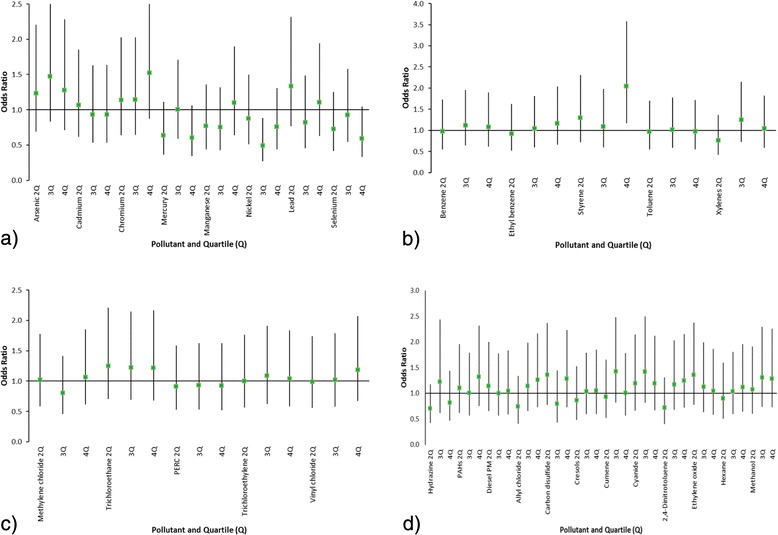
**a** Adjusted odds ratios with 95 % CI for interviewed ASD cases (*n* = 217) and controls (*n* = 224) associated with quartiles of exposure during pregnancy for metals. **b** Adjusted odds ratios with 95 % CI for interviewed ASD cases (*n* = 217) and controls (*n* = 224) associated with quartiles of exposure during pregnancy for aromatic solvents. **c** Adjusted odds ratios with 95 % CI for interviewed ASD cases (*n* = 217) and controls (*n* = 224) associated with quartiles of exposure during pregnancy for chlorinated solvents. **d** Adjusted odds ratios with 95 % CI for interviewed ASD cases (*n* = 217) and controls (*n* = 224) associated with quartiles of exposure during pregnancy for other HAPs

**Fig. 2:**
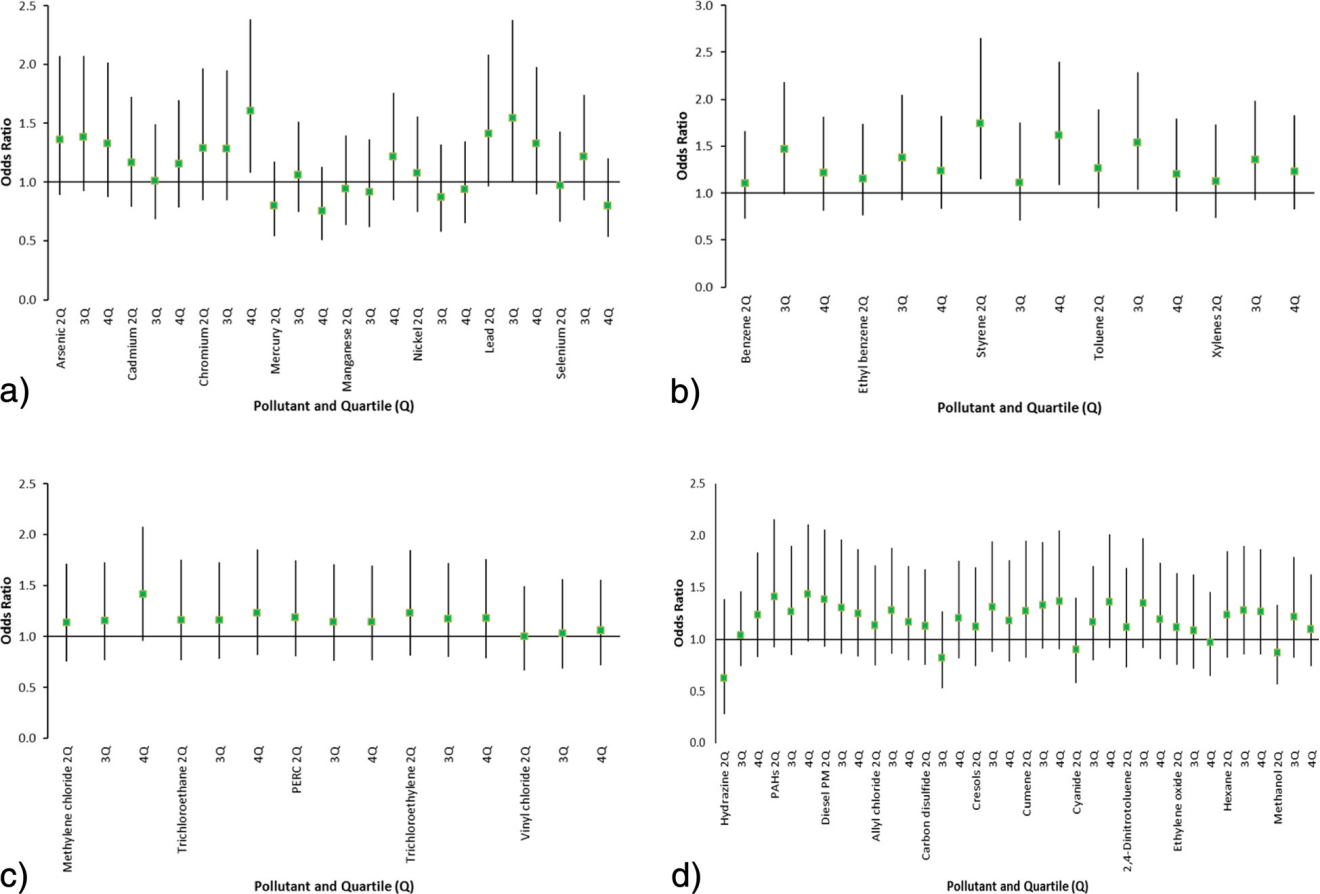
**a** Adjusted odds ratios with 95 % CI for ASD cases (*n* = 215) and BC controls (*n* = 4,856) associated with quartiles of exposure during pregnancy for metals. **b** Adjusted odds ratios with 95 % CI for ASD cases (*n* = 215) and BC controls (*n* = 4,856) associated with quartiles of exposure during pregnancy for aromatic solvents. **c** Adjusted odds ratios with 95 % CI for ASD cases (*n* = 215) and BC controls (*n* = 4,856) associated with quartiles of exposure during pregnancy for chlorinated solvents. **d** Adjusted odds ratios with 95 % CI for ASD cases (*n* = 215) and BC controls (*n* = 4,856) associated with quartiles of exposure during pregnancy for other HAPs

The adjusted odds ratios for the NATA air toxics grouped into quartiles of exposure during pregnancy with the three highest quartiles compared to the first quartile are shown in Figs. 1a-d and 2a-d and Table 3. For the interviewed case–control analyses, only styrene quartile four compared to quartile one remained significant after adjustment for covariates. Thus, women living in areas of the highest quartile of styrene exposure during pregnancy had a 2.04 (95 % CI = 1.17–3.58) odds of having an ASD diagnosed child than women living in areas of the lowest quartile of styrene during pregnancy, after adjusting for mother’s age, race, education, smoking, child’s year of birth, and child’s sex. Additional file 1: Table S1 provides the information on associations of NATA compounds for pregnancy, first year, and second year time periods for the interviewed cases and controls. The association with styrene was the only statistically significant associations of all NATA compounds for these periods. During the child’s first year of life, the fourth quartile of styrene compared to the first quartile resulted in a significantly elevated odds of ASD (OR = 1.86, 95 % CI = 1.07–3.25). During year two, the OR was elevated but not significant.

**Table 3:** Adjusted OR with 95 % CI for ASD associated with quartiles of exposure during pregnancy: interviewed cases (*n* = 217) and controls (*n* = 224) vs cases (*n* = 215) and birth certificate (BC) controls (*n* = 4856)^a^

Pollutant	Quartile (compared to 1)	Interviewed Control Adjusted				BC Control Adjusted			
		OR	Lower 95 % CI	Upper 95 % CI	p-value	OR	Lower 95 % CI	Upper 95 % CI	p-value
Metals									
Arsenic	2	1.23	0.69	2.20	0.477	1.36	0.89	2.07	0.154
	3	1.47	0.83	2.59	0.183	1.38	0.92	2.07	0.116
	4	1.28	0.71	2.28	0.415	1.33	0.87	2.02	0.183
Cadmium	2	1.07	0.61	1.86	0.817	1.17	0.79	1.73	0.443
	3	0.93	0.53	1.63	0.799	1.01	0.68	1.49	0.967
	4	0.93	0.53	1.64	0.804	1.15	0.78	1.70	0.466
Chromium	2	1.14	0.64	2.03	0.664	1.29	0.84	1.96	0.240
	3	1.14	0.64	2.03	0.651	1.28	0.85	1.95	0.240
	4	1.52	0.87	2.66	0.139	1.60	1.08	2.38	0.020
Mercury	2	0.64	0.36	1.11	0.112	0.79	0.54	1.17	0.250
	3	1.01	0.59	1.71	0.986	1.06	0.74	1.51	0.744
	4	0.60	0.34	1.06	0.079	0.75	0.51	1.13	0.169
Manganese	2	0.77	0.44	1.36	0.370	0.94	0.64	1.40	0.770
	3	0.75	0.43	1.32	0.321	0.92	0.62	1.36	0.665
	4	1.10	0.64	1.90	0.733	1.22	0.84	1.76	0.295
Nickel	2	0.87	0.51	1.50	0.622	1.08	0.74	1.56	0.700
	3	0.49	0.27	0.89	0.018	0.87	0.57	1.32	0.516
	4	0.76	0.44	1.31	0.317	0.93	0.65	1.35	0.718
Lead	2	1.33	0.77	2.32	0.311	1.41	0.96	2.08	0.080
	3	0.82	0.45	1.49	0.515	1.54	1.00	2.38	0.049
	4	1.10	0.63	1.94	0.734	1.33	0.90	1.97	0.158
Selenium	2	0.72	0.42	1.25	0.247	0.97	0.66	1.43	0.880
	3	0.93	0.54	1.58	0.776	1.21	0.85	1.74	0.292
	4	0.59	0.33	1.05	0.071	0.80	0.53	1.20	0.285
Aromatic Solvents									
Benzene	2	0.98	0.55	1.73	0.938	1.10	0.73	1.66	0.651
	3	1.12	0.64	1.95	0.690	1.46	0.98	2.18	0.059
	4	1.08	0.62	1.90	0.786	1.21	0.81	1.81	0.346
Ethyl benzene	2	0.92	0.52	1.62	0.763	1.15	0.76	1.74	0.498
	3	1.04	0.60	1.81	0.891	1.38	0.92	2.05	0.116
	4	1.16	0.66	2.03	0.609	1.23	0.84	1.82	0.291
Styrene	2	1.29	0.72	2.31	0.396	1.74	1.15	2.65	0.009
	3	1.09	0.60	1.98	0.784	1.11	0.71	1.75	0.648
	4	2.04	1.17	3.58	0.013	1.61	1.08	2.40	0.018
Toluene	2	0.97	0.55	1.70	0.914	1.26	0.84	1.90	0.262
	3	1.02	0.58	1.77	0.955	1.54	1.03	2.28	0.033
	4	0.98	0.55	1.72	0.930	1.20	0.80	1.79	0.376
Xylenes	2	0.76	0.42	1.36	0.352	1.12	0.73	1.73	0.593
	3	1.25	0.72	2.15	0.427	1.35	0.92	1.98	0.121
	4	1.04	0.59	1.83	0.895	1.23	0.83	1.83	0.310
Chlorinated Solvents									
Methylene chloride	2	1.02	0.58	1.78	0.958	1.13	0.75	1.71	0.548
	3	0.80	0.45	1.42	0.446	1.15	0.77	1.72	0.497
	4	1.07	0.61	1.85	0.822	1.41	0.96	2.07	0.082
Trichloroethane	2	1.25	0.71	2.21	0.447	1.16	0.77	1.75	0.485
	3	1.22	0.69	2.15	0.493	1.16	0.78	1.73	0.467
	4	1.22	0.68	2.17	0.509	1.23	0.82	1.85	0.323
Perchloroethylene	2	0.91	0.53	1.58	0.743	1.19	0.80	1.75	0.388
	3	0.93	0.53	1.62	0.791	1.14	0.76	1.71	0.530
	4	0.92	0.52	1.63	0.778	1.14	0.77	1.69	0.515
Trichloroethylene	2	1.00	0.57	1.76	0.996	1.23	0.81	1.85	0.331
	3	1.09	0.62	1.91	0.770	1.17	0.80	1.72	0.416
	4	1.04	0.59	1.84	0.902	1.18	0.79	1.76	0.423
Vinyl chloride	2	0.99	0.56	1.74	0.962	1.00	0.67	1.49	0.991
	3	1.02	0.58	1.79	0.950	1.03	0.68	1.56	0.879
	4	1.18	0.67	2.07	0.561	1.06	0.72	1.56	0.782
Other HAPs									
Hydrazine	2	0.71	0.42	1.17	0.178	0.62	0.28	1.39	0.248
	3	1.23	0.62	2.44	0.559	1.04	0.74	1.46	0.813
	4	0.82	0.47	1.44	0.492	1.23	0.83	1.84	0.304
PAHs	2	1.10	0.62	1.95	0.738	1.41	0.92	2.16	0.112
	3	1.01	0.57	1.79	0.973	1.27	0.85	1.90	0.251
	4	1.33	0.76	2.32	0.323	1.44	0.98	2.11	0.064
Diesel PM	2	1.15	0.66	2.00	0.634	1.38	0.93	2.06	0.112
	3	1.00	0.57	1.77	0.992	1.30	0.86	1.96	0.210
	4	1.04	0.59	1.84	0.887	1.25	0.83	1.87	0.282
Allyl chloride	2	0.75	0.41	1.34	0.328	1.13	0.75	1.71	0.548
	3	1.15	0.66	1.99	0.628	1.28	0.86	1.88	0.221
	4	1.26	0.73	2.17	0.405	1.17	0.80	1.70	0.424
Carbon disulfide	2	1.36	0.78	2.37	0.281	1.13	0.76	1.67	0.558
	3	0.79	0.44	1.45	0.451	0.82	0.53	1.27	0.371
	4	1.28	0.74	2.24	0.382	1.20	0.82	1.76	0.349
Cresols	2	0.86	0.49	1.53	0.611	1.12	0.74	1.69	0.591
	3	1.03	0.60	1.79	0.904	1.31	0.88	1.94	0.183
	4	1.05	0.60	1.85	0.854	1.18	0.79	1.76	0.424
Cumene	2	0.93	0.52	1.66	0.804	1.27	0.83	1.95	0.278
	3	1.43	0.82	2.48	0.205	1.33	0.91	1.93	0.143
	4	1.01	0.57	1.79	0.975	1.36	0.91	2.05	0.138
Cyanide	2	1.20	0.67	2.14	0.550	0.90	0.58	1.40	0.637
	3	1.42	0.81	2.49	0.218	1.17	0.80	1.71	0.430
	4	1.19	0.67	2.12	0.546	1.36	0.92	2.01	0.123
2,4-Dinitrotoluene	2	0.73	0.40	1.31	0.288	1.11	0.73	1.69	0.618
	3	1.18	0.68	2.03	0.566	1.35	0.92	1.98	0.130
	4	1.25	0.73	2.16	0.422	1.19	0.81	1.74	0.373
Ethylene Oxide	2	1.36	0.78	2.38	0.277	1.11	0.76	1.63	0.588
	3	1.13	0.64	2.00	0.686	1.08	0.72	1.62	0.710
	4	1.04	0.58	1.87	0.885	0.97	0.65	1.46	0.890
Hexane	2	0.90	0.51	1.60	0.718	1.23	0.82	1.85	0.313
	3	1.04	0.60	1.81	0.891	1.28	0.86	1.90	0.228
	4	1.12	0.64	1.96	0.689	1.27	0.86	1.87	0.239
Methanol	2	1.08	0.60	1.92	0.805	0.87	0.57	1.33	0.520
	3	1.31	0.74	2.29	0.353	1.21	0.82	1.79	0.328
	4	1.29	0.73	2.26	0.379	1.10	0.74	1.63	0.642
Index Scores									
Metals	2	0.69	0.39	1.23	0.209	0.96	0.65	1.42	0.842
	3	0.76	0.45	1.27	0.295	1.03	0.70	1.50	0.889
	4	0.93	0.53	1.63	0.790	1.17	0.80	1.70	0.423
Aromatic Solvents	2	0.81	0.47	1.40	0.447	1.17	0.79	1.73	0.446
	3	1.16	0.66	2.04	0.599	1.45	0.98	2.16	0.064
	4	1.10	0.63	1.93	0.745	1.20	0.81	1.78	0.368
Chlorinated Solvents	2	0.88	0.50	1.55	0.653	1.15	0.76	1.73	0.512
	3	1.00	0.58	1.73	1.000	1.10	0.75	1.62	0.609
	4	0.99	0.56	1.75	0.967	1.21	0.81	1.79	0.357

^a^All births, adjusted for mother’s age, education, race, smoking, child’s birth year (continuous), and child’s sex

As shown in Table 3 and Figs. 2a-d, there were significantly elevated odds ratios for both styrene and chromium for the BC control comparison. Styrene exhibited a significant effect comparing fourth to first quartile (OR = 1.61, 95 % CI = 1.08–2.40, *p* = 0.037). Chromium was also associated with significantly higher odds of having ASD (OR = 1.60, 95 % CI = 1.08–2.38, *p* = 0.018) when comparing the fourth to the first quartile of exposure, after adjustment for covariates. PAHs and methylene chloride were of borderline significance (*p* = 0.060 and *p* = 0.080, respectively). Lead exposure, third quartile compared to first, was significant (OR = 1.54, 95 % CI = 1.00–2.38) as well as toluene (OR = 1.54, 95 % CI = 1.03–2.28) third to first quartile. There was a borderline significant effect for benzene (OR = 1.46, 95 % CI = 0.98–2.18) third quartile compared to first. Table 3 also shows the overall measure of metal compounds (arsenic, cadmium, chromium, mercury, manganese, nickel, and lead), aromatic solvents (benzene, ethyl benzene, styrene, toluene, and xylenes), and chlorinated solvents (methylene chloride, trichloroethane, perchloroethylene, and vinyl chloride), none of which were statistically significant in either analysis. For the aromatic solvents, the third compared to first quartile was borderline (*p* = 0.064).

The supplement (Additional file 1: Tables S2 and S3, Figures S1-S4) provides information on the sensitivity analysis of the singleton births. The interviewed control, singleton-only analysis again resulted in only styrene exposure remaining significantly associated with ASD status after adjustment for maternal education, race, age, smoking, child’s year of birth, and child’s sex. For the BC control, singleton-only analysis, exposure to arsenic, methylene chloride, PAHs, lead and cyanide reached statistical significance in addition to chromium and styrene. Although the confidence intervals for these additional suspect pollutants were wide in the interviewed control analysis, the odds ratios were elevated to a similar degree.

We noted a possible differential response rate for cases and BC controls in Armstrong and Beaver counties (see Table 1), with fewer cases in Beaver and fewer controls for Armstrong. We conducted a sensitivity analysis for the second BC control analysis, excluding Beaver and Armstrong County cases and controls, to determine if there would be any effect on the results of the analyses. We found similar results for chromium (OR = 1.60, 95 % CI = 1.04 -2.47) and for styrene (OR = 1.70, 95 % CI = 1.12–2.58). The remaining compounds were not statistically significant in either the original or the sensitivity analysis excluding Armstrong and Beaver, indicating very little effect on the overall results from the study population in these counties.

Shown in Table 4 are the adjusted odds ratios and 95 % CIs for ASD (fourth compared to first quartile) associated with exposure to individual NATA air toxics that were statistically significant in either the interviewed case–control or the BC case–control analysis of all births or in the sensitivity analysis of only the singleton births. Seven compounds were found to be related to ASD risk (arsenic, chromium, methylene chloride, PAHs, styrene, cyanide and lead). There is consistency in the adjusted odds ratios for NATA compounds when comparing the results from the two different control groups. The most notable difference in the two control groups is the wider confidence intervals for the first control due to its smaller sample size.

**Table 4:** Adjusted^a^ OR (95 % CI) for ASD associated with exposure during pregnancy to NATA air toxics (fourth vs first quartile)

Pollutant	Interviewed (217 Cases and 224 Controls)		Birth Certificate (215 Cases and 4,856 Controls)	
	All Births	Singleton Births	All Births	Singleton Births
Arsenic	1.28 (0.71–2.28)	1.38 (0.75–2.52)	1.33 (0.87–2.02)	1.62 (1.03–2.54)^**^
Chromium	1.52 (0.87–2.66)	1.58 (0.88–2.81)	1.60 (1.08–2.38)^**^	1.65 (1.09–2.50)^**^
Methylene chloride	1.07 (0.61–1.85)	1.33 (0.74–2.36)	1.41 (0.96–2.07^)*^	1.63 (1.08–2.45)^**^
PAHs	1.33 (0.76–2.32)	1.56 (0.87–2.77)	1.44 (0.98–2.11)^*^	1.64 (1.09–2.46)^**^
Styrene	2.04 (1.17–3.58)^**^	2.23 (1.24–4.00)^**^	1.61 (1.08–2.40)^**^	1.67 (1.09–2.54)^**^
Lead	1.10 (0.63–1.94)	1.21 (0.67–2.18)	1.33 (0.90–1.97)	1.52 (1.00–2.30)^**^
Cyanide	1.19 (0.67–2.12)	1.32 (0.73–2.36)	1.36 (0.92–2.01)	1.52 (1.01–2.30)^**^

^a^Models adjusted for maternal smoking, age, education, race, child’s birth year, and sex of the child

^**^Significant (p-value < 0.05) ^*^ *p* < .10

A backward stepwise multiple logistic regression analysis was conducted in order to consider the contribution of these seven compounds identified as “compounds of interest” while controlling for important covariates of mother's education, race, age, smoking, child’s birth year and sex. For both the interviewed case–control and BC comparisons, only styrene remained in the final model of the seven air toxics, adjusting for covariates (fourth to first quartile, interviewed: OR = 2.04, 95 % CI = 1.17–3.58, *p* = 0.013; BC: OR = 1.61, 95 % CI = 1.08–2.40, *p* = 0.018).

Finally, a factor analysis using PCA and varimax rotation produced seven factors that explained almost 75 % of the variance of the original set of 30 air toxics. These factors appeared to represent pollution sources such as traffic, metal manufacturing, and plastic and rubber production. It is therefore possible that styrene or chromium, both individually associated with ASD risk in our study, are surrogates for related groups of agents. Future work will investigate methods to assess whether any of these groups of air toxics are associated with increased risk for ASD.

## Discussion

Although this study was exploratory in nature, we targeted air toxics found to be associated with ASD in three previous epidemiology studies using NATA [17–19]. Of the twelve compounds found to be associated with ASD in these previous three investigations, five compounds (arsenic, lead, chromium, styrene and methylene chloride) were found to be statistically significant in at least one of our four sets of control comparisons (interviewed all births, BC all births, interviewed singletons, and BC singletons). Styrene was statistically significant across all four comparisons, and chromium was statistically significant for all births and singleton births within the BC control adjusted analyses. Of the remaining 18 compounds examined, four exhibited statistical significance for the fourth compared to the first quartile or the third compared to the first quartile in at least one of the analyses. PAHs and cyanide exhibited elevated odds ratios comparing the fourth to the first quartile of exposure, with confidence limits that did not include one in the singleton BC comparisons. In the singleton BC analysis, benzene and toluene also exhibited statistically significant ORs in comparisons of the third to the first quartile, and diesel PM for the second to the first quartile. The odds ratios for a number of other compounds we evaluated were also elevated, but the confidence intervals included one. There was consistency in the magnitude of the adjusted odds ratios for NATA compounds when comparing the results based on the two different control groups within our study for the seven compounds found to be related to ASD risk (arsenic, chromium, methylene chloride, styrene, lead, cyanide and PAHs). The most notable difference in the two control groups is the wider confidence interval for the first control due to its smaller sample size resulting in lower power to detect small differences. Styrene and chromium showed the strongest estimates of risk for both case–control comparisons with a range of odds ratios of 1.61–2.23 for styrene and 1.52–1.65 for chromium in the four sets of case–control comparisons.

This study used a semi-ecological design. Personal risk factors were obtained at the individual level, but exposure to the air toxics was assessed at the group level (census tract level). Also, we applied the NATA concentration estimates for 2005 to the full time period of this study, and they may not have been representative of exposures for other years. In the interviewed analysis, although we had information on changes in residential addresses in the prenatal and postnatal period, we did not have explicit mobility patterns for the mother-child pairs outside of these residential census tracts.

Each control group analysis had its unique strengths and weaknesses. We were able to conduct maternal interviews for cases and for the first control group to obtain full residential history information, thereby decreasing exposure misclassification bias. We were also able to examine the association between ASD and estimated exposure to NATA air toxics not only during pregnancy, but also for the first and second years of life—the first study to do so. The second control group, because it was a random sample representative of all births in the six county area (weighted 4:1 male:female), removed the issue of response bias among the controls. However, a limitation of the second set of case–control analyses was the inability to capture residences during pregnancy other than the address reported at the time of birth. We noted that 16.1 % of the case mothers and 12.9 % of the interviewed control mothers reported moving to a new address during the year before the infant’s birth. Based on the sociodemographic characteristics of the birth certificate controls, the percentage that moved during pregnancy may have been even higher among the birth certificate controls, leading to exposure misclassification for the second set of control analyses.

Of the four studies (including ours) that used NATA to assess the risk of ASD associated with air toxics, the Windham et al. and Kalkbrenner et al. studies are the most similar with regard to case selection, and the use of birth certificates for residence and covariates. They identified cases using an existing surveillance system for California [19] and North Carolina and West Virginia [17]. Cases of ASD were identified as diagnosed through age 8. Windham et al. matched 284 cases by sex and month of birth to 657 controls from the same area randomly selected from the California birth–infant death certificate file; whereas Kalkbrenner et al. identified 383 children with ASD and 2,829 control children with speech and language impairment. Both used NATA chemicals from the 1996 HAP database as potential risk factors for ASD, which were assigned by census tract of the birth residence. In Windham’s investigation, because concentrations of many of the chemicals were highly correlated, chemicals were combined into mechanistic and structural groups calculating summary index scores. The adjusted odds ratios (AORS) were elevated by 50 % in the top quartile of chlorinated solvents (95 % CI = 1.08–2.23) (methylene chloride, trichloroethylene, and vinyl chloride) and heavy metals (95 % CI = 1.05 to 2.12) (nickel, cadmium and mercury) compared to the combined lower two quartiles in cases compared to controls [19]. Kalkbrenner et al. calculated odds ratios corresponding to the odds of ASD for high pollutant concentrations (80^th^ percentile) compared to low pollutant concentrations (20^th^ percentile). Hazardous air pollutants with more precise and elevated OR estimates included methylene chloride, quinoline and styrene, with odds ratios that were between 1.4 and 1.8 [17]. Results of these two studies suggested a potential association between ASD and metals and possibly solvents.

Our interviewed case–control group was most like the Roberts et al. investigation as neither study utilized a surveillance system to identify ASD cases and both relied on personal interview for residential history of the mother during pregnancy and afterwards and other potential risk factors. Roberts et al. was able to identify the offspring of mothers who participated in the Nurses’ Health Study II between 1987 and 2002 diagnosed with ASD [18]. Roberts et al. compared a total of 325 ASD cases to 22,098 controls from the same birth cohort matched on age and gender. Although ASD diagnosis was not confirmed, the authors did administer a diagnostic test by telephone to a randomly selected subset, and the medical background of the participants most likely adds strength to the accuracy of the diagnosis. We obtained diagnostic test results from the psychologist or health care provider of record. Roberts et al., comparing the highest quintile of exposure to the lowest for both sexes, found significant associations between ASD and exposure to overall metals (OR = 1.6, 95 % CI = 1.1–2.4), lead (OR = 1.6, 95 % CI = 1.1–2.3), manganese (OR = 1.5, 95 % CI = 1.1–2.2), mercury (OR = 1.4, 95 % CI = 0.9–2.0), nickel (OR = 1.7, 95 % CI = 1.1–2.5) and methylene chloride (OR = 1.5, 95 % CI = 1.0–2.1). Other HAPS that reached significance in the highest quintile were cadmium and diesel PM. They adjusted for possible confounding factors including socioeconomic level, census tract level SES measures, maternal age, and year of birth [18].

Our second control was similar to Windham’s investigation because residence and personal risk factors were obtained from the birth certificate in both studies. In general, the four NATA studies noted increased risk of ASD for certain heavy metals, chlorinated solvents (methylene chloride) and specific aromatic solvents (styrene).

Each previous NATA study investigated a different geographical area and used NATA assessments from varying years. Windham et al. and Kalkbrenner et al. used the 1996 NATA assessment, while our present study used the most recent NATA data available (2005). Roberts assigned exposure using the assessment closest to each year of birth, so all assessments from 1990 to 2002 were included. The US EPA advises that NATA results from different years should not be compared since there are changes in the methodology and emissions or source inputs for each assessment [20].

Nevertheless, we compiled the results for 13 NATA compounds for cases and controls from each of the major NATA/autism studies [17–19] for comparison purposes (see Additional file 1: Table S4). In general, the means and standard deviations in the Windham study using 1996 NATA reflect higher estimates of exposure for 9 of the 13 compounds. These levels were higher than those in our study for many of the metals (chromium, lead, mercury, and nickel), as well as for benzene, diesel PM, methylene chloride, styrene, and trichloroethylene. It should be noted that Windham’s population was from the San Francisco Bay area, which is also highly urban and industrial and reflects air toxics modeled for 1996. In our study (SW PA), the arsenic and cadmium mean levels were higher compared to the other three studies. With respect to styrene, which was significantly associated with ASD in our study using either control group, the levels in the four studies ranked (from highest to lowest): Windham et al. (100 ng/m^3^ cases, 90 ng/m^3^ controls), Roberts et al. (60 ng/m^3^), our present study (39 ng/m^3^ cases, 38 ng/m^3^ controls), and Kalkbrenner et al. (23 ng/m^3^ North Carolina, 10 ng/m^3^ West Virginia). The exposures for the West Virginia participants in the Kalkbrenner study tended to be the lowest, most likely because West Virginia is more rural compared to the regions considered in the other investigations. Both Roberts and Kalkbrenner found relationships with ASD and styrene exposure. Styrene was not entered as an individual pollutant in any of Windham’s models.

Current research points to a variety of cellular, molecular and inflammatory pathways that directly damage brain structure or lead to a predisposition for disease that affects the CNS system through neuroinflammation, oxidative stress and glial activation [23, 24]. Diodovich et al. noted that styrene has been shown to cross the placenta. Animal studies clearly support the role of styrene in promoting cell proliferation and cell cycle progression as potentially favoring alterations in gene expression [25].

Styrene emissions come from a variety of sources, including point sources (e.g. plastic manufacturing, hazardous waste treatment, paint and coating manufacturing, and organic chemical manufacturing facilities) and on-road sources such as car exhaust [26, 27]. According to NATA 2005 county-level estimates, 4–30 % of styrene emissions across our six county study area were attributed to point sources. Non-point sources contributed 13–77 %, and on-road sources contributed 14–49 %. Other possible sources of styrene include occupational exposures and cigarette smoke [28]. Maternal smoking was considered in our analysis.

Chromium is also emitted from a variety of sources, processes, and industries, such as coal and oil combustion, electroplating, leather tanning, cement-producing plants, tobacco smoke, and the metal and textile industries [29]. Chromium is an additive in steel production as a hardener and a primary component of stainless steel. In our study area, point sources contributed 50–83 % of chromium emissions, non-point sources 7–22 %, and mobile sources <1 %.

## Conclusions

Results of this case–control study in Southwestern Pennsylvania suggest that living in areas with higher ambient levels of styrene and chromium is associated with increased risk of ASD; moreover, there are borderline effects for PAHs and methylene chloride. When considering singleton births only, there were additional significant associations with arsenic, lead and cyanide. Our study was unique in that we were able to utilize two different kinds of control group analyses: the first using personal interviews capturing the period of pregnancy to age two, and the second a random sample of all births (weighted by sex) from the same time period based on birth certificate information. Our dataset enabled us to adjust for other suspected risk factors of ASD (age of mother, smoking history, maternal education, and race).

The above compounds represent air toxics of interest which require greater scrutiny to determine individual risk beyond the census tract or group level. Ground level monitoring of these targeted compounds should be conducted for multiple time periods (days of week and hours of the day) at multiple locations, including around major industrial sources. Future work should also include time activity patterns to obtain spatiotemporal estimates of exposure to air toxics, taking into account the dynamic movement of individuals during daily life. In addition, a national or statewide autism registry would enhance access to a greater proportion of all ASD cases and enable greater participation into future research.

## Additional file

Additional file 1: Recruitment flowcharts and results of the singleton only analysis. (DOCX 294 kb)

## Abbreviations

BC: Birth certificate
CI: Confidence interval
GIS: Geographic information system
NATA: National Air Toxics Assessment
OR: Odds ratio
PM: Particulate matter
