# Supplementary material for: Air toxics and the risk of autism spectrum disorder: the results of a population based case–control study in southwestern Pennsylvania
Source: Environ Health. 2015 Oct 6;14:80. doi: 10.1186/s12940-015-0064-1 (PMC4596286; doi:10.1186/s12940-015-0064-1)
Supplement: Additional file 1: — Recruitment flowcharts and results of the singleton only analysis. (DOCX 294 kb) [file 12940_2015_64_MOESM1_ESM.docx]

**Supplemental Material**

**Title:** Air Toxics and the Risk of Autism Spectrum Disorder: The Results of a Population Based Case Control Study in Southwestern Pennsylvania

**Authors:** Talbott EO, Marshall LP, Rager JR, Arena V, Sharma RK, Stacy SL

**Table of Contents**

Supplemental Material, Figure S1. Case recruitment flowchart

Supplemental Material, Figure S2. Interviewed control recruitment flowchart

Supplemental Material, Table S1. Adjusted odds ratios with 95% CI for ASD associated with quartiles of exposure during the full pregnancy and years 1 and 2 of life, adjusted for mother’s age, race, education, smoking, child’s birth year, and sex of the child: interviewed cases (n=217) vs controls (n=224)

Supplemental Material, Table S2: Characteristics of ASD Cases and Controls (singleton births only)

Supplemental Material, Table S3. Adjusted odds ratios with 95% CI for ASD associated with quartiles of exposure during pregnancy: cases (n=199) vs first (interviewed) control (n=215) and second (birth certificate) control (n=4,670), single pollutant (singleton births only)

Supplemental Material, Figure S3a. Metals: Adjusted odds ratios with 95% CI for ASD associated with quartiles of exposure during pregnancy: interviewed cases (n=199) vs controls (n=215) (singleton births only).

Supplemental Material, Figure S3b. Figure S1b. Metals: Adjusted odds ratios with 95% CI for ASD associated with quartiles of exposure during pregnancy: cases (n=197) vs controls (n=4.670) using birth certificate information (singleton births only).

Supplemental Material, Figure S4a. Aromatic solvents: Adjusted odds ratios with 95% CI for ASD associated with quartiles of exposure during pregnancy: interviewed cases (n=199) vs controls (n=215) (singleton births only).

Supplemental Material, Figure S4b. Figure S2b. Aromatic solvents: Adjusted odds ratios with 95% CI for ASD associated with quartiles of exposure during pregnancy: cases (n=197) vs controls (n=4.670) using birth certificate information (singleton births only).

Supplemental Material, Figure S5a. Chlorinated solvents: Adjusted odds ratios with 95% CI for ASD associated with quartiles of exposure during pregnancy: interviewed cases (n=199) vs controls (n=215) (singleton births only).

Supplemental Material, Figure S5b. Chlorinated solvents: Adjusted odds ratios with 95% CI for ASD associated with quartiles of exposure during pregnancy: cases (n=197) vs controls (n=4.670) using birth certificate information (singleton births only).

Supplemental Material, Figure S6a. Other HAPs: Adjusted odds ratios with 95% CI for ASD associated with quartiles of exposure during pregnancy: interviewed cases (n=199) vs controls (n=215) (singleton births only).

Supplemental Material, Figure S6b. Other HAPs: Adjusted odds ratios with 95% CI for ASD associated with quartiles of exposure during pregnancy: cases (n=197) vs controls (n=4.670) using birth certificate information (singleton births only).

Supplemental Material, Table S4. Comparison of exposure estimates (means and standard deviations) to NATA air toxics between major epidemiology studies of ASD and air toxics (ng/m^3^)

**Figure S1.** Case recruitment flowchart

^*^Excluding 3 duplicates

^**^1 missing reason ineligible

**Figure S2.** Interviewed control recruitment flowchart

**Table S1.** Adjusted odds ratios with 95% CI for ASD associated with quartiles of exposure during the full pregnancy and years 1 and 2 of life, adjusted for mother’s age, race, education, smoking, child’s birth year, and sex of the child: interviewed cases (n=217) vs controls (n=224)

| **Pollutant**  **Metals** |  | **Full Pregnancy** | **1^st^ Year of Life** | **2^nd^ Year of Life** |
| --- | --- | --- | --- | --- |
| Arsenic compounds | 2  3  4 | 1.23 (0.69, 2.20)  1.47 (0.83, 2.59)  1.28 (0.71, 2.28) | 1.32 (0.74, 2.37)  1.50 (0.85, 2.64)  1.33 (0.74, 2.38) | 1.17 (0.65, 2.09)  1.26 (0.72, 2.22)  1.19 (0.66, 2.15) |
| Cadmium compounds | 2  3  4 | 1.07 (0.61, 1.86)  0.93 (0.53, 1.63)  0.93 (0.53, 1.64) | 0.98 (0.56, 1.71)  1.10 (0.62, 1.92)  1.03 (0.58, 1.81) | 0.77 (0.44, 1.33)  0.93 (0.53, 1.63)  0.83 (0.47, 1.47) |
| Chromium compounds | 2  3  4 | 1.14 (0.64, 2.03)  1.14 (0.64, 2.03)  1.52 (0.87, 2.66) | 1.22 (0.68, 2.18)  1.18 (0.66, 2.10)  1.64 (0.94, 2.87) | 1.52 (0.86, 2.69)  1.24 (0.69, 2.21)  1.59 (0.91, 2.77) |
| Mercury compounds | 2  3  4 | 0.64 (0.36, 1.11)  1.01 (0.59, 1.71)  0.60 (0.34, 1.06) | 0.65 (0.37, 1.13)  1.03 (0.60, 1.76)  0.63 (0.35, 1.12) | 0.57 (0.33, 1.00)  1.05 (0.61, 1.79)  0.60 (0.34, 1.06) |
| Manganese compounds | 2  3  4 | 0.77 (0.44, 1.36)  0.75 (0.43, 1.32)  1.10 (0.64, 1.90) | 0.87 (0.50, 1.51)  0.65 (0.37, 1.14)  1.19 (0.69, 2.04) | 0.80 (0.46, 1.41)  0.73 (0.42, 1.26)  1.10 (0.64, 1.89) |
| Nickel compounds | 2  3  4 | 0.87 (0.51, 1.50)  **0.49 (0.27, 0.89)**  0.76 (0.44, 1.31) | 0.90 (0.53, 1.55)  0.52 (0.29, 0.95)  0.84 (0.49, 1.44) | 0.84 (0.49, 1.43)  0.54 (0.30, 0.97)  0.82 (0.47, 1.41) |
| Lead compounds | 2  3  4 | 1.33 (0.77, 2.32)  0.82 (0.45, 1.49)  1.10 (0.63, 1.94) | 1.06 (0.61, 1.85)  0.85 (0.47, 1.54)  0.94 (0.53, 1.64) | 1.15 (0.66, 2.00)  0.94 (0.52, 1.69)  0.95 (0.54, 1.67) |
| Selenium compounds | 2  3  4 | 0.72 (0.42, 1.25)  0.93 (0.54, 1.58)  0.59 (0.33, 1.05) | 0.64 (0.37, 1.10)  0.88 (0.51, 1.51)  0.67 (0.38, 1.18) | 0.58 (0.33, 1.00)  0.85 (0.50, 1.46)  0.55 (0.31, 0.98) |
| **Aromatic Solvents** | | | | |
| Benzene | 2  3  4 | 0.98 (0.55, 1.73)  1.12 (0.64, 1.95)  1.08 (0.62, 1.90) | 1.05 (0.60, 1.85)  1.25 (0.72, 2.19)  1.20 (0.69, 2.09) | 0.83 (0.48, 1.45)  1.13 (0.64, 1.98)  1.01 (0.57, 1.76) |
| Ethyl benzene | 2  3  4 | 0.92 (0.52, 1.62)  1.04 (0.60, 1.81)  1.16 (0.66, 2.03) | 1.20 (0.68, 2.12)  1.12 (0.64, 1.94)  1.29 (0.74, 2.26) | 1.22 (0.69, 2.16)  1.12 (0.65, 1.94)  1.15 (0.65, 2.01) |
| Styrene | 2  3  4 | 1.29 (0.72, 2.31)  1.09 (0.60, 1.98)  **2.04 (1.17, 3.58)** | 1.08 (0.61, 1.94)  1.10 (0.61, 1.98)  **1.86 (1.07, 3.25)** | 1.09 (0.61, 1.94)  1.05 (0.58, 1.89)  1.59 (0.91, 2.79) |
| Toluene | 2  3  4 | 0.97 (0.55, 1.70)  1.02 (0.58, 1.77)  0.98 (0.55, 1.72) | 1.22 (0.70, 2.12)  1.12 (0.64, 1.96)  1.14 (0.65, 2.00) | 0.92 (0.53, 1.59)  1.00 (0.57, 1.76)  1.01 (0.57, 1.77) |
| Xylenes  (isomers and mixture) | 2  3  4 | 0.76 (0.42, 1.36)  1.25 (0.72, 2.15)  1.04 (0.59, 1.83) | 0.87 (0.49, 1.55)  1.32 (0.77, 2.29)  1.12 (0.64, 1.98) | 0.80 (0.45, 1.42)  1.27 (0.73, 2.20)  1.04 (0.59, 1.85) |

| **Pollutant**  **Chlorinated Solvents** |  | **Full Pregnancy** | **1^st^ Year of Life** | **2^nd^ Year of Life** |
| --- | --- | --- | --- | --- |
| Methylene chloride | 2  3  4 | 1.02 (0.58, 1.78)  0.80 (0.45, 1.42)  1.07 (0.61, 1.85) | 1.18 (0.67, 2.05)  0.99 (0.56, 1.75)  1.28 (0.73, 2.23) | 0.94 (0.54, 1.62)  0.96 (0.54, 1.68)  0.98 (0.56, 1.71) |
| Trichloroethane | 2  3  4 | 1.25 (0.71, 2.21)  1.22 (0.69, 2.15)  1.22 (0.68, 2.17) | 1.07 (0.61, 1.89)  1.33 (0.76, 2.34)  1.18 (0.66, 2.09) | 0.99 (0.57, 1.74)  1.19 (0.68 , 2.10)  1.04 (0.59, 1.84) |
| Perchloroethylene | 2  3  4 | 0.91 (0.53, 1.58)  0.93 (0.53, 1.62)  0.92 (0.52, 1.63) | 0.83 (0.48, 1.43)  0.91 (0.52, 1.62)  0.92 (0.53, 1.61) | 0.81 (0.47, 1.39)  0.79 (0.44, 1.41)  0.91 (0.52, 1.59) |
| Trichloroethylene | 2  3  4 | 1.00 (0.57, 1.76)  1.09 (0.62, 1.91)  1.04 (0.59, 1.84) | 1.10 (0.62, 1.92)  1.11 (0.63, 1.97)  1.20 (0.68, 2.13) | 0.88 (0.50, 1.53)  0.97 (0.55, 1.71)  1.13 (0.64, 1.97) |
| Vinyl chloride | 2  3  4 | 0.99 (0.56, 1.74)  1.02 (0.58, 1.79)  1.18 (0.67, 2.07) | 1.12 (0.64, 1.97)  1.09 (0.62, 1.92)  1.38 (0.79, 2.42) | 1.10 (0.63, 1.93)  1.16 (0.66, 2.02)  1.33 (0.76, 2.32) |
| **Other HAPs** | | | | |
| Hydrazine | 2  3  4 | 0.71 (0.42, 1.17)  1.23 (0.62, 2.44)  0.82 (0.47, 1.44) | 0.88 (0.53, 1.45)  1.21 (0.62, 2.39)  0.95 (0.54, 1.66) | 0.64 (0.38, 1.05)  1.02 (0.52, 2.02)  0.85 (0.49, 1.48) |
| PAHs | 2  3  4 | 1.10 (0.62, 1.95)  1.01 (0.57, 1.79)  1.33 (0.76, 2.32) | 0.95 (0.54, 1.67)  1.06 (0.60, 1.88)  1.24 (0.71, 2.17) | 0.95 (0.54, 1.67)  1.08 (0.62, 1.89)  1.14 (0.65, 2.02) |
| Diesel Particulate Matter | 2  3  4 | 1.15 (0.66, 2.00)  1.00 (0.57, 1.77)  1.04 (0.59, 1.84) | 1.29 (0.74, 2.25)  1.21 (0.68, 2.16)  1.11 (0.63, 1.96) | 0.97 (0.56, 1.69)  1.08 (0.62, 1.88)  0.89 (0.51, 1.58) |
| Allyl chloride | 2  3  4 | 0.75 (0.41, 1.34)  1.15 (0.66, 1.99)  1.26 (0.73, 2.17) | 0.91 (0.50, 1.63)  1.17 (0.67, 2.01)  1.24 (0.72, 2.13) | 0.81 (0.44, 1.47)  1.11 (0.64, 1.92)  1.16 (0.68, 1.98) |
| Carbon disulfide | 2  3  4 | 1.36 (0.78, 2.37)  0.79 (0.44, 1.45)  1.28 (0.74, 2.24) | 1.24 (0.71, 2.16)  0.79 (0.44, 1.43)  1.25 (0.71, 2.19) | 1.09 (0.62, 1.89)  0.81 (0.45, 1.45)  0.99 (0.56, 1.75) |
| Cresols/  Cresylic acid | 2  3  4 | 0.86 (0.49, 1.53)  1.03 (0.60, 1.79)  1.05 (0.60, 1.85) | 1.03 (0.59, 1.82)  1.18 (0.69, 2.04)  1.27 (0.72, 2.22) | 0.66 (0.37, 1.16)  1.07 (0.62, 1.85)  1.00 (0.58, 1.75) |
| Cumene | 2  3  4 | 0.93 (0.52, 1.66)  1.43 (0.82, 2.48)  1.01 (0.57, 1.79) | 0.83 (0.47, 1.47)  1.51 (0.87, 2.61)  0.99 (0.56, 1.75) | 0.77 (0.43, 1.37)  1.70 (0.99, 2.92)  0.77 (0.43, 1.39) |
| Cyanide | 2  3  4 | 1.20 (0.67, 2.14)  1.42 (0.81, 2.49)  1.19 (0.67, 2.12) | 1.07 (0.60, 1.91)  1.43 (0.82, 2.49)  1.24 (0.70, 2.20) | 0.97 (0.54, 1.73)  1.57 (0.90, 2.75)  1.11 (0.63, 1.99) |
| 2,4-Dinitrotoluene | 2  3  4 | 0.73 (0.40, 1.31)  1.18 (0.68, 2.03)  1.25 (0.73, 2.16) | 0.81 (0.45, 1.48)  1.12 (0.65, 1.93)  1.22 (0.71, 2.09) | 0.77 (0.42, 1.40)  1.14 (0.66, 1.96)  1.17 (0.68, 2.00) |
| Ethylene Oxide | 2  3  4 | 1.36 (0.78, 2.38)  1.13 (0.64, 2.00)  1.04 (0.58, 1.87) | 1.08 (0.62, 1.87)  1.05 (0.60, 1.86)  0.94 (0.53, 1.68) | 1.06 (0.61, 1.83)  1.02 (0.57, 1.81)  0.85 (0.48, 1.52) |
| Hexane | 2  3  4 | 0.90 (0.51, 1.60)  1.04 (0.60, 1.81)  1.12 (0.64, 1.96) | 1.08 (0.61, 1.91)  1.29 (0.74, 2.25)  1.22 (0.70, 2.13) | 0.89 (0.50, 1.57)  1.26 (0.73, 2.19)  1.02 (0.58, 1.79) |
| Methanol | 2  3  4 | 1.08 (0.60, 1.92)  1.31 (0.74, 2.29)  1.29 (0.73, 2.26) | 0.88 (0.50, 1.54)  0.82 (0.49, 1.38)  1.05 (0.60, 1.84) | 1.08 (0.61, 1.91)  1.19 (0.68, 2.07)  1.12 (0.64, 1.93) |

**Table S1 (continued)**

| **Pollutant**  **Index Scores** |  | **Full Pregnancy** | **1^st^ Year of Life** | **2^nd^ Year of Life** |
| --- | --- | --- | --- | --- |
| Metals  (without Se) | 2  3  4 | 0.69 (0.39, 1.23)  0.75 (0.45, 1.27)  0.93 (0.53, 1.63) | 0.88 (0.50, 1.54)  0.82 (0.49, 1.38)  1.05 (0.60, 1.84) | 0.90 (0.51, 1.58)  0.82 (0.49, 1.39)  0.98 (0.56, 1.74) |
| Aromatic Solvents | 2  3  4 | 0.81 (0.47, 1.40)  1.16 (0.66, 2.04)  1.10 (0.63, 1.93) | 0.96 (0.56, 1.65)  1.33 (0.75, 2.35)  1.20 (0.68, 2.09) | 0.88 (0.52, 1.51)  1.20 (0.68, 2.13)  1.10 (0.62, 1.92) |
| Chlorinated Solvents  (with  1,1,1-Trichloroethane) | 2  3  4 | 0.88 (0.50, 1.55)  1.00 (0.58, 1.73)  0.99 (0.56, 1.75) | 1.01 (0.57, 1.79)  1.12 (0.65, 1.93)  1.15 (0.65, 2.03) | 0.96 (0.54, 1.68)  1.14 (0.67, 1.96)  1.03 (0.58, 1.82) |

**Table S1 (continued)**

**Table S2.** Characteristics of ASD Cases and Controls (singleton births only)

| **Characteristic** | **Cases Interviewed^*^**  **(n=199)** | **Controls**  **Interviewed^*^**  **(n=217)** | **Cases**  **Birth Certificate^**^**  **(n=198)** | **Controls**  **Birth Certificate^**^**  **(n=4,782)** |
| --- | --- | --- | --- | --- |
|  | N (%) | N (%) | N (%) | N (%) |
| Gender:  Male  Female | 155 (77.9)  44 (22.1) | 170 (78.3)  47 (21.7) | 154 (77.8)  44 (22.2) | 3,839 (80.3)  943 (19.7) |
| Year of birth – n(%)  2005  2006  2007  2008  2009 | 40 (20.1)  52 (26.1)  45 (22.6)  34 (17.1)  28 (14.1) | 53 (24.4)  41 (18.9)  45 (20.7)  37 (17.1)  41 (18.9) | 40 (20.2)  52 (26.3)  45 (22.7)  33 (16.7)  28 (14.1) | 1,117 (23.4)  1,105 (23.1)  1,030 (21.5)  847 (17.7)  683 (14.3) |
| County at birth – n(%)  Allegheny  Armstrong  Beaver  Butler  Washington  Westmoreland | 122 (61.3)  7 (3.5)  7 (3.5)  13 (6.5)  20 (10.1)  30 (15.1) | 128 (59.0)  2 (0.9)  18 (8.3)  17 (7.8)  22 (10.1)  30 (13.8) | 120 (60.6)  7 (3.5)  7 (3.5)  14 (7.1)  19 (9.6)  31 (15.7) | 2,777 (58.1)  145 (3.0)  378 (7.9)  387 (8.1)  433 (9.1)  662 (13.8) |
| Maternal age – mean (SD)^1^ | 30.3 (5.3) | 31.7 (4.7) | 30.4 (5.3) | 28.4 (6.0) |
| Paternal age – mean (SD)^2,10^ | 32.5 (6.06) | 33.6 (6.0) | 32.7 (5.9) | 31.4 (6.6) |
| Mother race^3^  White  Black  Other | 176 (88.4)  15 (7.5)  2 (1.0) | 210 (96.8)  4 (1.8)  2 (0.9) | 178 (89.9)  16 (8.1)  4 (2.0) | 3,894 (81.7)  667 (14.0)  203 (4.3) |
| Mother’s education^4^  < High school graduate  High school graduate and some college  ≥ College graduate | 3 (1.5)  86 (43.2)  110 (55.3) | 2 (0.9)  44 (20.3)  171 (78.8) | 6 (3.0)  84 (42.4)  108 (54.5) | 461 (9.7)  2,446 (51.4)  1,853 (38.9) |
| Low birth weight (< 2500g)^5,8^ (based on birth certificate) | 12 (6.1) | 7 (3.2) | 12 (6.1) | 250 (5.2) |
| Preterm birth (< 37 weeks)^6,9^ (based on birth certificate) | 20 (10.3) | 15 (7.0) | 20 (10.3) | 413 (8.8) |
| Maternal Smoking during pregnancy or in 3 months  prior to pregnancy^7^  Yes  No | 50 (25.1)  149 (74.9) | 22 (10.1)  195 (89.9) | 27 (13.7)  170 (86.3) | 1,129 (24.1)  3,564 (75.9) |

^*^Interviewed information on case and control

^**^Information from birth certificate only

Birth certificate control missing information:

^1^Missing ? ^5^Missing ?

^2^Missing ? ^6^Missing ?

^3^Missing 18 ^7^Missing 1 case; 89 controls

^4^Missing 22

Interview missing information

^8^Missing 1

^9^Missing 9

^10^ Missing 6

| **Table S3.** Adjusted odds ratios with 95% CI for ASD associated with quartiles of exposure during pregnancy: cases (n=199) vs first (interviewed) control (n=215) and second (birth certificate) control (n=4,670), single pollutant (singleton births only)* | | | | | | | | | |
| --- | --- | --- | --- | --- | --- | --- | --- | --- | --- |
| **Pollutant** | **Quartile (compared to 1)** | **First Control Adjusted** | | | | **Second Control Adjusted** | | | |
| *Metals* |  | *OR* | *Lower 95% CI* | *Upper 95% CI* | *p* | *OR* | *Lower 95% CI* | *Upper 95% CI* | *p* |
| Arsenic | 2 | 1.12 | 0.61 | 2.05 | 0.716 | 1.52 | 0.96 | 2.42 | 0.075 |
|  | 3 | 1.64 | 0.91 | 2.93 | 0.098 | **1.72** | **1.11** | **2.67** | **0.015** |
|  | 4 | 1.38 | 0.75 | 2.52 | 0.301 | **1.62** | **1.03** | **2.54** | **0.037** |
| Cadmium | 2 | 1.21 | 0.68 | 2.15 | 0.512 | 1.28 | 0.85 | 1.95 | 0.242 |
|  | 3 | 0.96 | 0.53 | 1.72 | 0.884 | 1.14 | 0.75 | 1.73 | 0.536 |
|  | 4 | 1.14 | 0.63 | 2.05 | 0.662 | 1.33 | 0.88 | 2.00 | 0.173 |
| Chromium | 2 | 1.09 | 0.60 | 1.97 | 0.783 | 1.29 | 0.83 | 2.01 | 0.258 |
|  | 3 | 1.12 | 0.62 | 2.02 | 0.712 | 1.27 | 0.82 | 1.96 | 0.289 |
|  | 4 | 1.58 | 0.88 | 2.81 | 0.123 | **1.65** | **1.09** | **2.50** | **0.018** |
| Mercury | 2 | 0.69 | 0.39 | 1.23 | 0.213 | 0.90 | 0.60 | 1.35 | 0.622 |
|  | 3 | 1.07 | 0.62 | 1.85 | 0.819 | 1.11 | 0.76 | 1.61 | 0.588 |
|  | 4 | 0.64 | 0.35 | 1.14 | 0.128 | 0.79 | 0.52 | 1.21 | 0.281 |
| Manganese | 2 | 0.84 | 0.47 | 1.50 | 0.546 | 1.01 | 0.66 | 1.53 | 0.974 |
|  | 3 | 0.83 | 0.46 | 1.47 | 0.517 | 1.03 | 0.68 | 1.56 | 0.888 |
|  | 4 | 1.07 | 0.61 | 1.90 | 0.808 | 1.34 | 0.91 | 1.97 | 0.141 |
| Nickel | 2 | 0.82 | 0.47 | 1.43 | 0.481 | 1.15 | 0.78 | 1.70 | 0.482 |
|  | 3 | **0.48** | **0.26** | **0.88** | **0.018** | 0.92 | 0.59 | 1.43 | 0.722 |
|  | 4 | 0.84 | 0.48 | 1.48 | 0.550 | 1.06 | 0.72 | 1.55 | 0.774 |
| Lead | 2 | 1.33 | 0.75 | 2.37 | 0.325 | 1.50 | 0.99 | 2.27 | 0.056 |
|  | 3 | 0.84 | 0.46 | 1.56 | 0.586 | **1.68** | **1.06** | **2.65** | **0.027** |
|  | 4 | 1.21 | 0.67 | 2.18 | 0.520 | **1.52** | **1.00** | **2.30** | **0.049** |
| Selenium | 2 | 0.76 | 0.43 | 1.33 | 0.333 | 1.03 | 0.69 | 1.53 | 0.902 |
|  | 3 | 0.95 | 0.54 | 1.67 | 0.864 | 1.18 | 0.80 | 1.72 | 0.404 |
|  | 4 | 0.58 | 0.32 | 1.05 | 0.073 | 0.79 | 0.51 | 1.21 | 0.280 |
| *Aromatic Solvents* | | | | | | | | | |
| Benzene | 2 | 0.98 | 0.54 | 1.77 | 0.942 | 1.05 | 0.68 | 1.64 | 0.820 |
|  | 3 | 1.18 | 0.66 | 2.10 | 0.571 | **1.52** | **1.01** | **2.31** | **0.047** |
|  | 4 | 1.18 | 0.66 | 2.10 | 0.576 | 1.30 | 0.85 | 1.97 | 0.224 |
| Ethyl benzene | 2 | 0.91 | 0.51 | 1.65 | 0.765 | 1.25 | 0.81 | 1.93 | 0.320 |
|  | 3 | 1.09 | 0.61 | 1.94 | 0.781 | 1.44 | 0.95 | 2.20 | 0.088 |
|  | 4 | 1.29 | 0.72 | 2.31 | 0.385 | 1.38 | 0.92 | 2.08 | 0.122 |
| Styrene | 2 | 1.43 | 0.79 | 2.60 | 0.238 | **1.85** | **1.19** | **2.88** | **0.006** |
|  | 3 | 0.94 | 0.50 | 1.78 | 0.861 | 1.16 | 0.72 | 1.88 | 0.537 |
|  | 4 | **2.23** | **1.24** | **4.00** | **0.007** | **1.67** | **1.09** | **2.54** | **0.018** |
| Toluene | 2 | 0.93 | 0.52 | 1.67 | 0.812 | 1.18 | 0.76 | 1.83 | 0.457 |
|  | 3 | 1.11 | 0.62 | 1.98 | 0.720 | **1.61** | **1.07** | **2.44** | **0.023** |
|  | 4 | 1.06 | 0.59 | 1.90 | 0.846 | 1.28 | 0.84 | 1.95 | 0.244 |
| Xylenes | 2 | 0.79 | 0.43 | 1.45 | 0.451 | 1.26 | 0.80 | 1.99 | 0.319 |
|  | 3 | 1.37 | 0.78 | 2.42 | 0.277 | 1.50 | 1.00 | 2.25 | 0.053 |
|  | 4 | 1.19 | 0.66 | 2.13 | 0.562 | 1.41 | 0.93 | 2.15 | 0.109 |

| **Pollutant** | | **Quartile (compared to 1)** | **First Control Adjusted** | | | | **Second Control Adjusted** | | | |
| --- | --- | --- | --- | --- | --- | --- | --- | --- | --- | --- |
| *Chlorinated Solvents* | |  | *OR* | *Lower*  *95% CI* | *Upper*  *95% CI* | *p-value* | *OR* | *Lower*  *95% CI* | *Upper*  *95% CI* | *p-value* |
| Methylene chloride | | 2 | 1.17 | 0.65 | 2.09 | 0.596 | 1.23 | 0.79 | 1.91 | 0.356 |
|  |  | 3 | 0.84 | 0.46 | 1.52 | 0.565 | 1.26 | 0.82 | 1.94 | 0.298 |
|  |  | 4 | 1.33 | 0.74 | 2.36 | 0.340 | **1.63** | **1.08** | **2.45** | **0.020** |
| Trichloroethane | | 2 | 1.22 | 0.67 | 2.21 | 0.514 | 1.25 | 0.80 | 1.95 | 0.318 |
|  |  | 3 | 1.25 | 0.70 | 2.24 | 0.451 | 1.27 | 0.83 | 1.94 | 0.268 |
|  |  | 4 | 1.37 | 0.75 | 2.50 | 0.300 | 1.40 | 0.91 | 2.16 | 0.126 |
| Perchloroethylene | | 2 | 0.82 | 0.46 | 1.46 | 0.508 | 1.04 | 0.69 | 1.57 | 0.859 |
|  |  | 3 | 0.95 | 0.53 | 1.69 | 0.854 | 1.16 | 0.77 | 1.77 | 0.474 |
|  |  | 4 | 0.94 | 0.53 | 1.69 | 0.841 | 1.17 | 0.78 | 1.76 | 0.444 |
| Trichloroethylene | | 2 | 1.21 | 0.67 | 2.18 | 0.526 | 1.46 | 0.94 | 2.26 | 0.089 |
|  |  | 3 | 1.29 | 0.71 | 2.32 | 0.401 | 1.37 | 0.90 | 2.07 | 0.138 |
|  |  | 4 | 1.35 | 0.74 | 2.45 | 0.327 | 1.41 | 0.92 | 2.16 | 0.118 |
| Vinyl chloride | | 2 | 1.04 | 0.57 | 1.87 | 0.906 | 0.99 | 0.65 | 1.50 | 0.951 |
|  |  | 3 | 1.11 | 0.62 | 1.98 | 0.733 | 1.09 | 0.71 | 1.67 | 0.701 |
|  |  | 4 | 1.14 | 0.64 | 2.04 | 0.660 | 1.06 | 0.70 | 1.59 | 0.791 |
| *Other HAPs* | | | | | | | | | | |
| Hydrazine | | 2 | 0.72 | 0.43 | 1.23 | 0.233 | 0.72 | 0.32 | 1.62 | 0.429 |
|  |  | 3 | 1.41 | 0.69 | 2.90 | 0.350 | 1.08 | 0.75 | 1.54 | 0.690 |
|  |  | 4 | 0.83 | 0.47 | 1.49 | 0.538 | 1.31 | 0.86 | 1.99 | 0.208 |
| PAHs | | 2 | 1.18 | 0.65 | 2.14 | 0.581 | **1.58** | **1.01** | **2.48** | **0.047** |
|  |  | 3 | 1.13 | 0.62 | 2.06 | 0.702 | 1.40 | 0.91 | 2.16 | 0.125 |
|  |  | 4 | 1.56 | 0.87 | 2.77 | 0.133 | **1.64** | **1.09** | **2.46** | **0.018** |
| Diesel PM | | 2 | 1.23 | 0.69 | 2.20 | 0.478 | **1.56** | **1.02** | **2.39** | **0.040** |
|  |  | 3 | 1.06 | 0.59 | 1.93 | 0.838 | 1.49 | 0.96 | 2.30 | 0.076 |
|  |  | 4 | 1.23 | 0.69 | 2.22 | 0.486 | 1.42 | 0.92 | 2.18 | 0.111 |
| Allyl chloride | | 2 | 0.71 | 0.39 | 1.30 | 0.265 | 1.12 | 0.73 | 1.72 | 0.602 |
|  |  | 3 | 1.00 | 0.57 | 1.76 | 1.000 | 1.26 | 0.84 | 1.89 | 0.267 |
|  |  | 4 | 1.15 | 0.66 | 2.02 | 0.622 | 1.16 | 0.78 | 1.72 | 0.468 |
| Carbon disulfide | | 2 | 1.37 | 0.77 | 2.44 | 0.288 | 1.18 | 0.78 | 1.80 | 0.432 |
|  |  | 3 | 0.90 | 0.48 | 1.66 | 0.724 | 0.90 | 0.57 | 1.41 | 0.647 |
|  |  | 4 | 1.31 | 0.74 | 2.33 | 0.351 | 1.24 | 0.83 | 1.86 | 0.291 |
| Cresols | | 2 | 0.87 | 0.48 | 1.58 | 0.642 | 1.08 | 0.70 | 1.68 | 0.721 |
|  |  | 3 | 1.09 | 0.62 | 1.93 | 0.757 | 1.37 | 0.91 | 2.08 | 0.133 |
|  |  | 4 | 1.10 | 0.62 | 1.97 | 0.741 | 1.20 | 0.79 | 1.83 | 0.399 |
| Cumene | | 2 | 1.07 | 0.59 | 1.93 | 0.834 | 1.48 | 0.95 | 2.32 | 0.082 |
|  |  | 3 | 1.44 | 0.81 | 2.55 | 0.210 | 1.41 | 0.94 | 2.11 | 0.095 |
|  |  | 4 | 1.18 | 0.65 | 2.14 | 0.576 | 1.44 | 0.93 | 2.22 | 0.103 |
| Cyanide | | 2 | 1.08 | 0.59 | 1.98 | 0.795 | 0.93 | 0.58 | 1.49 | 0.768 |
|  |  | 3 | 1.39 | 0.78 | 2.47 | 0.268 | 1.24 | 0.83 | 1.86 | 0.290 |
|  |  | 4 | 1.32 | 0.73 | 2.36 | 0.355 | **1.52** | **1.01** | **2.30** | **0.044** |
| 2,4-Dinitrotoluene | | 2 | 0.71 | 0.39 | 1.30 | 0.265 | 1.15 | 0.75 | 1.76 | 0.531 |
|  |  | 3 | 1.01 | 0.57 | 1.78 | 0.971 | 1.28 | 0.86 | 1.92 | 0.228 |
|  |  | 4 | 1.14 | 0.65 | 2.01 | 0.646 | 1.18 | 0.79 | 1.75 | 0.422 |
| Ethylene Oxide | | 2 | 1.27 | 0.71 | 2.27 | 0.427 | 1.15 | 0.76 | 1.74 | 0.508 |
|  |  | 3 | 1.27 | 0.70 | 2.29 | 0.435 | 1.26 | 0.82 | 1.93 | 0.294 |
|  |  | 4 | 1.10 | 0.60 | 2.00 | 0.766 | 1.11 | 0.73 | 1.70 | 0.626 |
| Hexane | | 2 | 0.87 | 0.48 | 1.57 | 0.639 | 1.31 | 0.84 | 2.02 | 0.230 |
|  |  | 3 | 1.05 | 0.59 | 1.87 | 0.857 | 1.46 | 0.96 | 2.23 | 0.075 |
|  |  | 4 | 1.20 | 0.68 | 2.13 | 0.533 | 1.45 | 0.96 | 2.19 | 0.079 |
| Methanol | | 2 | 0.95 | 0.52 | 1.74 | 0.874 | 0.87 | 0.55 | 1.37 | 0.538 |
|  |  | 3 | 1.43 | 0.80 | 2.56 | 0.233 | 1.35 | 0.90 | 2.03 | 0.152 |
|  |  | 4 | 1.45 | 0.81 | 2.59 | 0.211 | 1.23 | 0.81 | 1.86 | 0.328 |
|  | ^*^Adjusted for mother’s age, education, race, smoking, child’s birth year (continuous), child’s sex | | | | | | | | |  |

**Figure S3a.** *Metals:* Adjusted odds ratios with 95% CI for ASD associated with quartiles of exposure during pregnancy: interviewed cases (n=199) vs controls (n=215) (singleton births only)


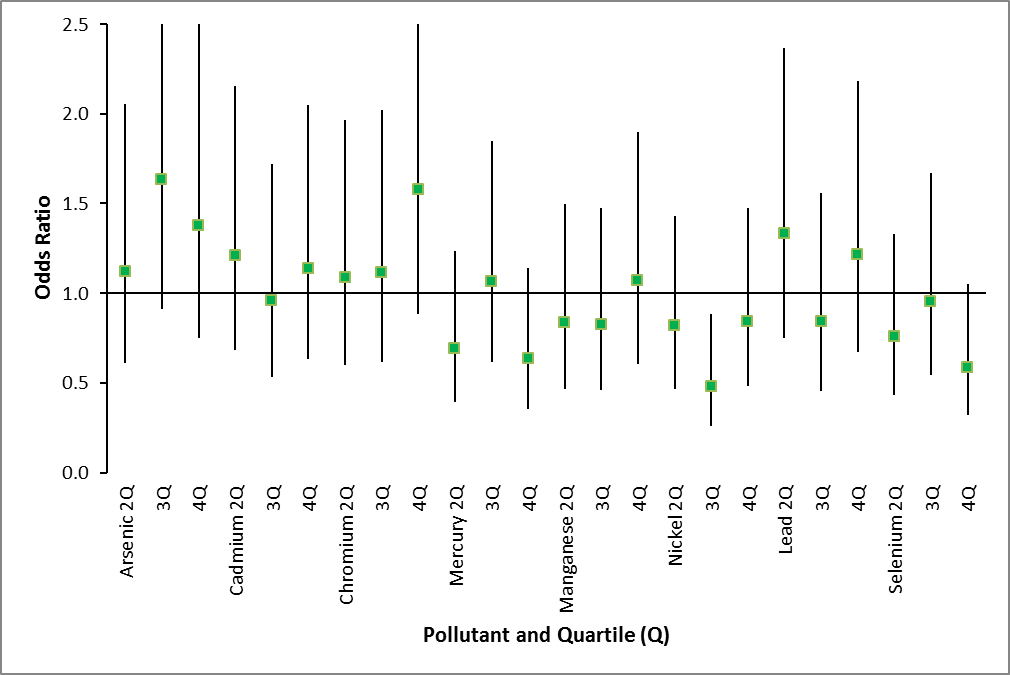


**Figure S3b.** *Metals:* Adjusted odds ratios with 95% CI for ASD associated with quartiles of exposure during pregnancy: cases (n=197) vs controls (n=4.670) using birth certificate information (singleton births only)

**
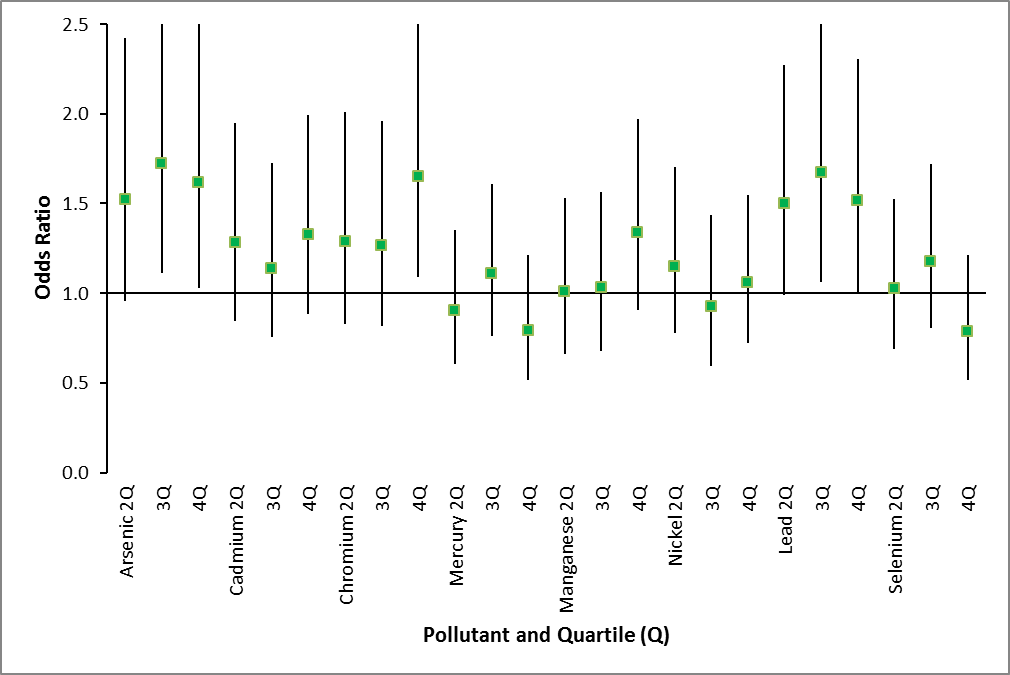
**

**Figure S4a.** *Aromatic solvents:* Adjusted odds ratios with 95% CI for ASD associated with quartiles of exposure during pregnancy: interviewed cases (n=199) vs controls (n=215) (singleton births only)


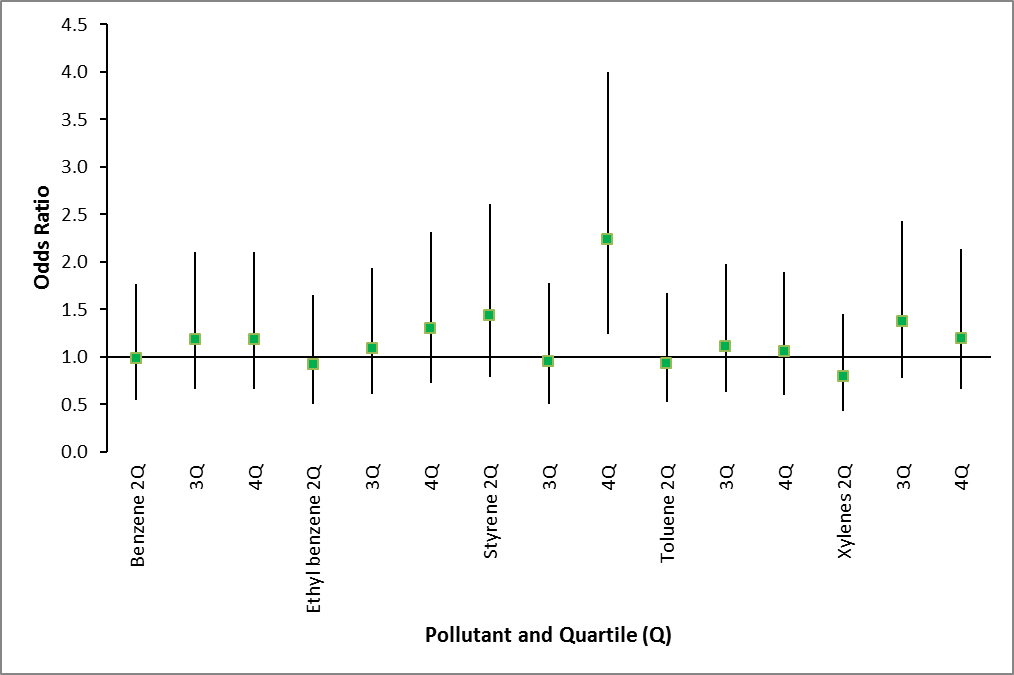


**Figure S4b.** *Aromatic solvents:* Adjusted odds ratios with 95% CI for ASD associated with quartiles of exposure during pregnancy: cases (n=197) vs controls (n=4.670) using birth certificate information (singleton births only)


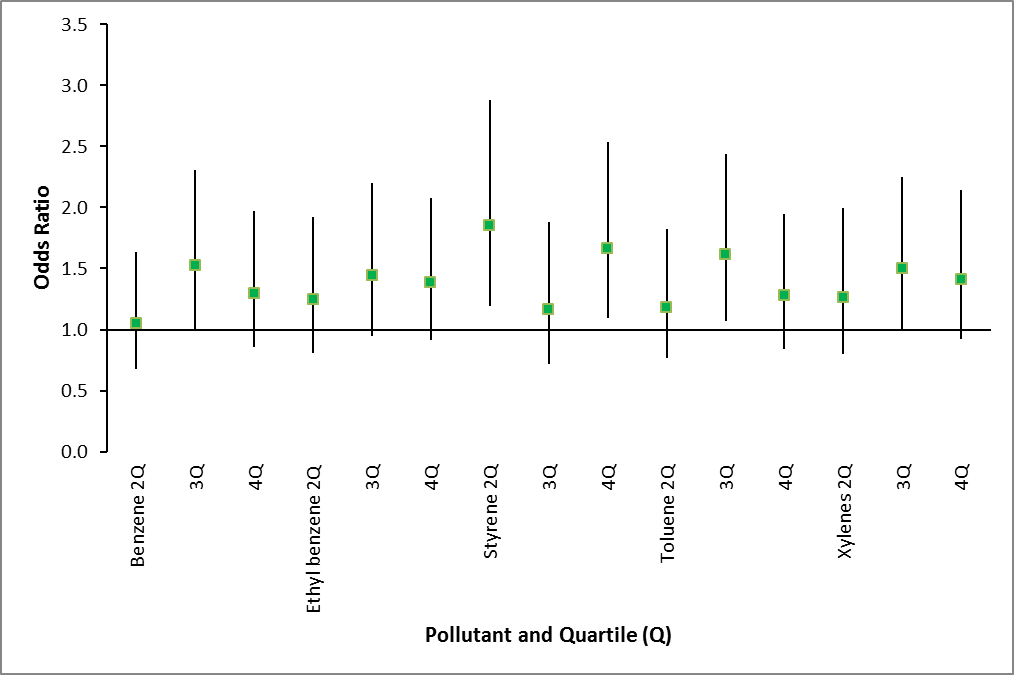


**Figure S5a.** *Chlorinated solvents:* Adjusted odds ratios with 95% CI for ASD associated with quartiles of exposure during pregnancy: interviewed cases (n=199) vs controls (n=215) (singleton births only)


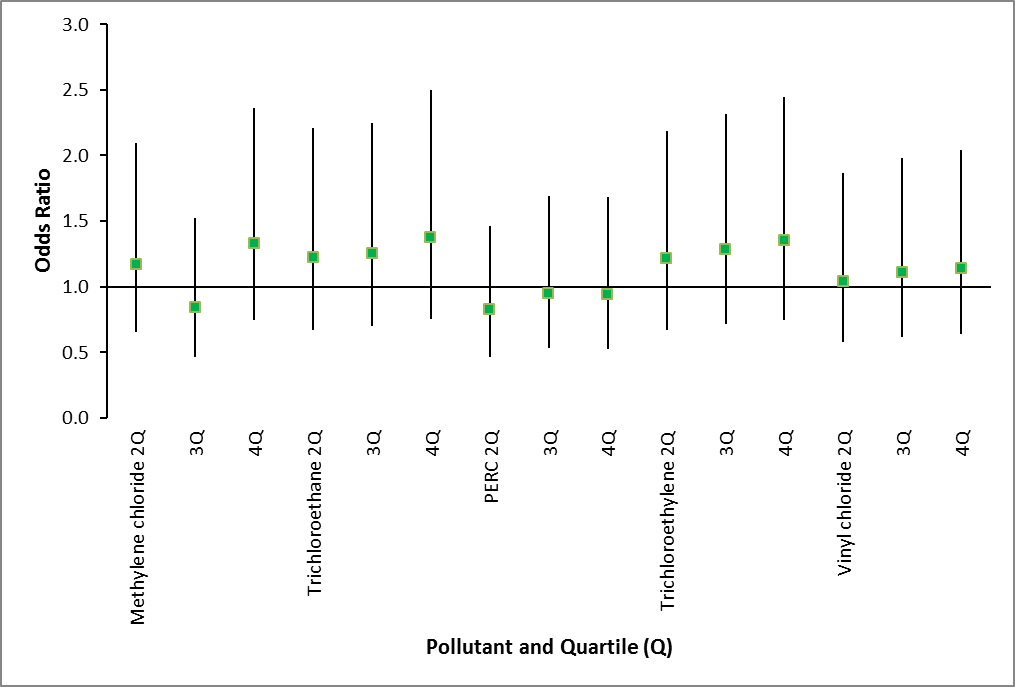


**Figure S5b.** *Chlorinated solvents:* Adjusted odds ratios with 95% CI for ASD associated with quartiles of exposure during pregnancy: cases (n=197) vs controls (n=4.670) using birth certificate information (singleton births only)

**
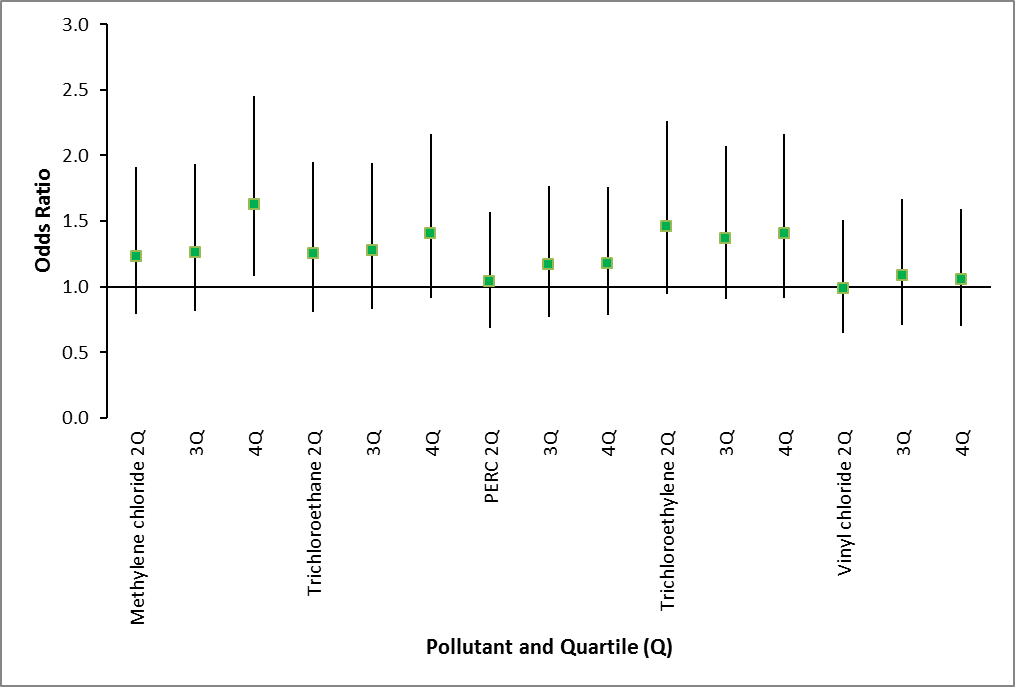
**

**Figure S6a.** *Other HAPs:* Adjusted odds ratios with 95% CI for ASD associated with quartiles of exposure during pregnancy: interviewed cases (n=199) vs controls (n=215) (singleton births only)


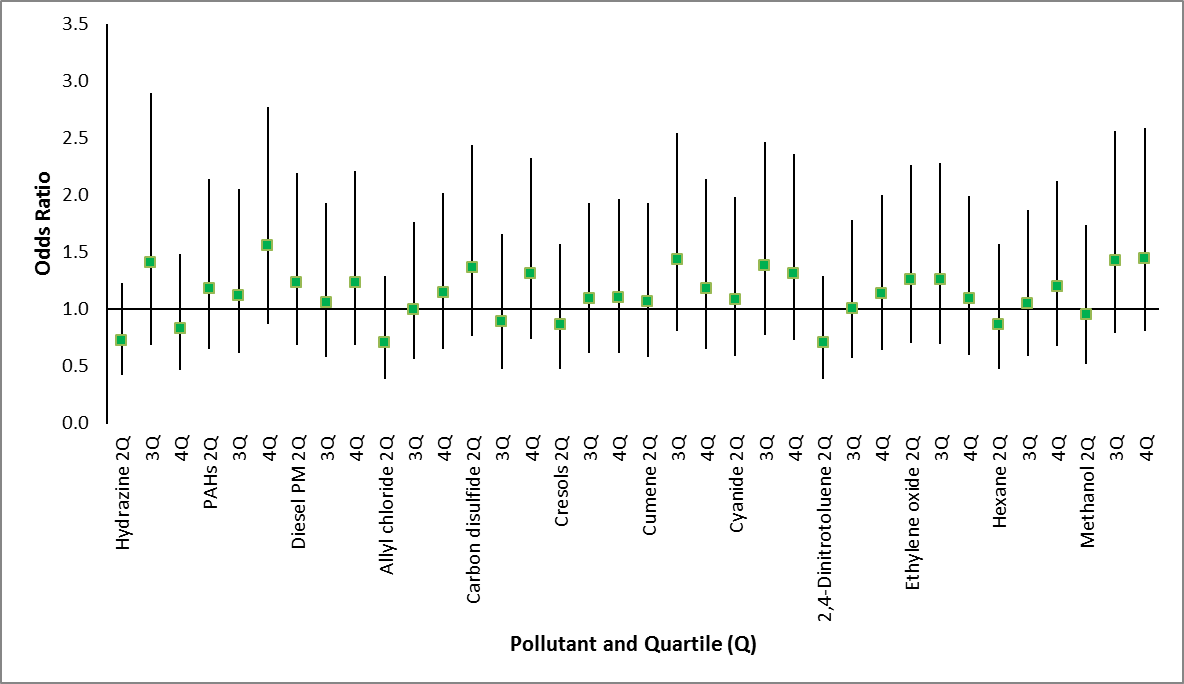


**Figure S6b.** *Other HAPs:* Adjusted odds ratios with 95% CI for ASD associated with quartiles of exposure during pregnancy: cases (n=197) vs controls (n=4.670) using birth certificate information (singleton births only)


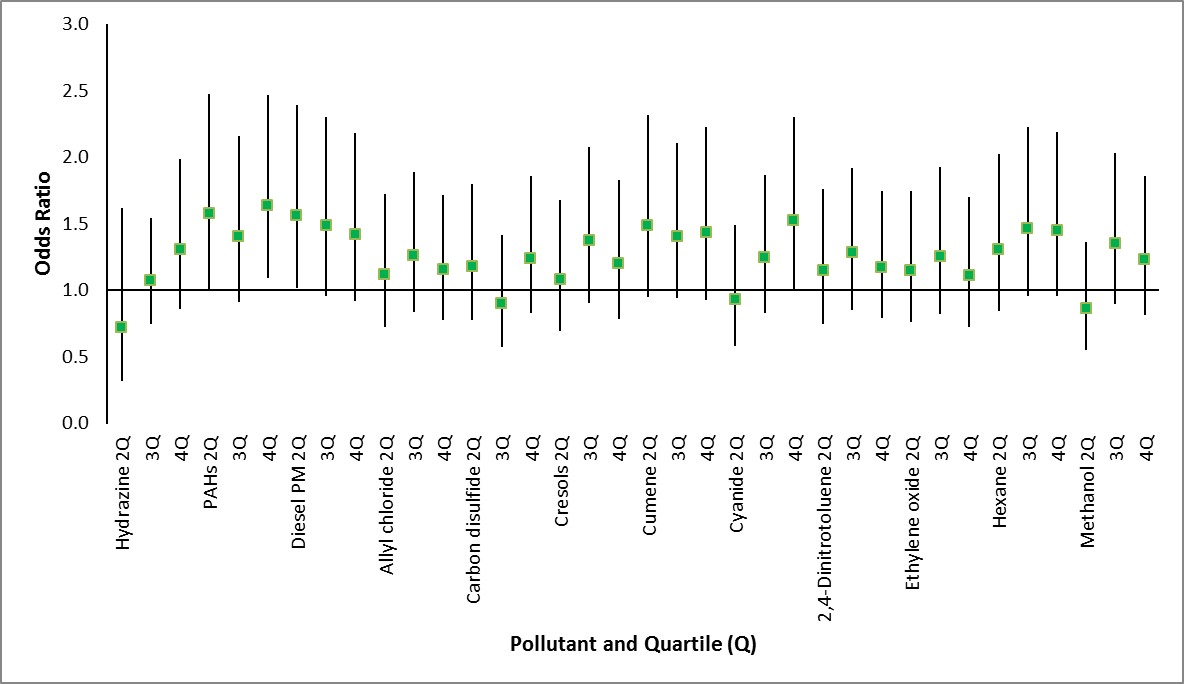
**Table S4.** Comparison of exposure estimates (means and standard deviations) to NATA air toxics between major epidemiology studies of ASD and air toxics (ng/m^3^)

|  | **Present Study** | | **Windham** | | **Kalkbrenner** | | **Roberts** |
| --- | --- | --- | --- | --- | --- | --- | --- |
| **Region** | Southwest Pennsylvania | | California | | North Carolina and West Virginia | | Nation-wide |
| **NATA year** | 2005  (ng/m^3^) | | 1996  (ng/m^3^) | | 1996  (ng/m^3^) | | 1990, 1996, 1999, 2002 (ng/m^3^) |
| **Sample size and statistic reported** | Cases:  N=217  **Mean (SD)** | Controls:  N=224  **Mean (SD)** | Cases:  N=284  **Mean (SD)** | Controls:  N=657  **Mean (SD)** | All NC:  N=1,733  **Mean (SD)^*^** | All WV:  N=1,096  **Mean (SD)^*^** | N=22,426  **Mean (SD)**  **Range** |
| **Metals:**  *Arsenic* | 1.21 (0.43) | 1.17 (0.36) | 0.1 (0.06) | 0.1 (0.05) | 0.15 (2.0) | 0.03 (6.3) | 0.2 (0.5)  0-50 |
| *Cadmium* | 0.17 (0.08) | 0.17 (0.08) | 0.1 (0.2) | 0.1 (0.1) | 0.058 (1.9) | 0.012 (3.8) | 0.2 (0.5)  0-30 |
| *Chromium* | 2.40 (2.91) | 2.63 (5.13) | 4.4 (5.7) | 3.9 (4.9) | 2.3 (2.8) | 0.1 (6.0) | 1 (2)  2*10^-3^-60 |
| *Lead* | 4.78 (6.56) | 4.67 (6.41) | 9.3 (11.8) | 8.2 (9.2) | 2.42 (1.9) | `0.29 (3.7) | 5 (10)  0-650 |
| *Manganese* | 2.70 (4.58) | 2.45 (3.82) | 3.2 (1.7) | 3.2 (1.6) | 5.2 (2.1) | 0.6 (4.9) | 4 (8)  0-620 |
| *Mercury* | 0.07 (0.05) | 0.08 (0.07) | 0.8 (1.9) | 0.6 (1.0) | 0.251 (1.6) | 0.060 (3.0) | 2 (1)  1*10^-3^-80 |
| *Nickel* | 1.27 (2.82) | 1.65 (5.70) | 4.3 (5.9) | 3.7 (3.8) | 1.1 (2.0) | 0.2 (6.3) | 5 (10)  0-420 |
| **Aromatics:**  *Benzene* | 1087.71 (291.73) | 1065.71  (271.72) | 1710 (620) | 1660 (500) | 1209.7 (1.3) | 845.6 (1.4) | Not reported |
| *Styrene* | 38.80 (20.42) | 37.64 (40.45) | 100 (60 ) | 90 (50) | 23.4 (2.3) | 9.6 (4.2) | 60 (100)  0-7300 |
| **Chlorinated solvents:**  *Trichloro-*  *ethylene* | 79.32 (20.28) | 78.00 (27.15) | 190 (110) | 170 (80) | 118.8 (1.3) | 90.3 (1.2) | 300 (300)  5-5000 |
| *Vinyl*  *chloride* | 0.13 (0.18) | 0.13 (0.24) | 20 (60) | 10 (20) | 1.05 (2.3) | 0.32 (4.1) | 20 (60)  0-3200 |
| **Other HAPs:**  *Diesel PM* | 513.64 (419.50) | 474.16 (382.39) | 3370 (3480) | 2890 (2350) | 1497 (1.3) | 1241 (1.8) | 1900 (3000)  0-139,800 |
| *Methylene*  *chloride* | 259.21 (39.23) | 258.84 (36.17) | 680 (480) | 640 (350) | 539.8 (1.9) | 202.3 (1.3) | 400 (700)  0.6-419,000 |
| *Geometric mean and standard deviation | | | | | | | |
